# The Hepatitis B Virus Nucleocapsid—Dynamic Compartment for Infectious Virus Production and New Antiviral Target

**DOI:** 10.3390/biomedicines9111577

**Published:** 2021-10-29

**Authors:** Matthias Niklasch, Peter Zimmermann, Michael Nassal

**Affiliations:** Internal Medicine II/Molecular Biology, University Hospital Freiburg, Hugstetter Str. 55, D-79106 Freiburg, Germany; matthias.niklasch@uniklinik-freiburg.de (M.N.); peter.zimmermann@uniklinik-freiburg.de (P.Z.)

**Keywords:** capsid, capsid assembly, capsid assembly modulator (CAM), capsid protein, chronic hepatitis B (CHB), combination therapy, core protein, core protein allosteric modulator (CpAM), HBcAg, HBeAg, HBV, HBV cure

## Abstract

Hepatitis B virus (HBV) is a small enveloped DNA virus which replicates its tiny 3.2 kb genome by reverse transcription inside an icosahedral nucleocapsid, formed by a single ~180 amino acid capsid, or core, protein (Cp). HBV causes chronic hepatitis B (CHB), a severe liver disease responsible for nearly a million deaths each year. Most of HBV’s only seven primary gene products are multifunctional. Though less obvious than for the multi-domain polymerase, P protein, this is equally crucial for Cp with its multiple roles in the viral life-cycle. Cp provides a stable genome container during extracellular phases, allows for directed intracellular genome transport and timely release from the capsid, and subsequent assembly of new nucleocapsids around P protein and the pregenomic (pg) RNA, forming a distinct compartment for reverse transcription. These opposing features are enabled by dynamic post-transcriptional modifications of Cp which result in dynamic structural alterations. Their perturbation by capsid assembly modulators (CAMs) is a promising new antiviral concept. CAMs inappropriately accelerate assembly and/or distort the capsid shell. We summarize the functional, biochemical, and structural dynamics of Cp, and discuss the therapeutic potential of CAMs based on clinical data. Presently, CAMs appear as a valuable addition but not a substitute for existing therapies. However, as part of rational combination therapies CAMs may bring the ambitious goal of a cure for CHB closer to reality.

## 1. Introduction

**Figure 1:**
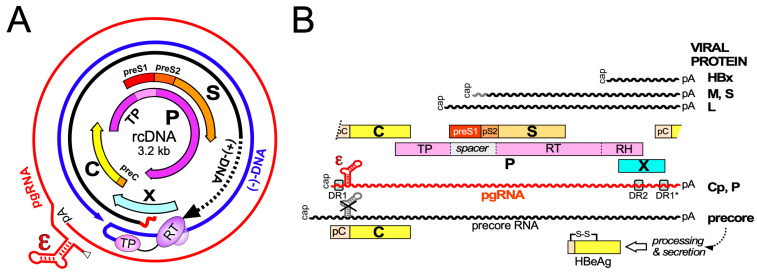
HBV genome organization, transcription, and translation. (**A**) Genome organization. The blue and the black line depict rcDNA with the 5′ terminally linked TP domain of P protein on the minus-strand and the RNA primer (red) on the plus-strand; the dashed black line symbolizes the incompletely filled-in 3′end. Colored internal arrows represent the ORFs with their respective preS1, preS2, and preC extensions. The outer red line depicts pgRNA with the 5′ proximal ε signal. Note that the actual transcription template is cccDNA, not rcDNA. (**B**) Viral transcripts and protein products. RNAs are shown above (subgenomic) and below (greater-than-genome length) a linear representation of the ORFs as present on the terminally redundant pgRNA; DR1, DR1*, and DR2 are direct repeats involved in rcDNA formation. As is common for eukaryotes, the most cap-proximal ORF on each RNA is translated, for precore protein enabled by the inclusion of the preC start codon in the 5′ extension; precore processing at both termini and secretion yield HBeAg (see Section 3.2). Translation of pgRNA yields Cp from the first and P protein from the second ORF; the latter initiation mechanism is still unclear.

Hepatitis B virus (HBV), the etiological agent of acute and chronic hepatitis B (CHB) in humans, is a hepatotropic small enveloped DNA virus that replicates through reverse transcription. Although its ~3 kb genome encodes only seven primary gene products (Figure 1) HBV is one of the most successful human pathogens. According to World Health Organization (WHO) estimates two billion people carry antibodies indicating prior exposure to the virus, and close to 300 million have become chronic virus carriers [1]. They are at a high risk to develop severe liver disease such as fibrosis, cirrhosis, and primary liver cancer [2], with nearly a million deaths per year. HBV infection can be prevented by a prophylactic vaccine yet current treatments for chronic infection are usually not curative [3]. Owing to pronounced adverse effects, only a small fraction of patients are eligible for type-I interferon (IFN) based therapies, e.g., with pegylated IFN-α (pegIFNα), which after a finite 24- or 48-week treatment leads in ~10% of the patients to a sustained loss of hepatitis B surface antigen (HBsAg) [3]. Often the virus is controlled but not eliminated, a condition termed “functional cure”. Most patients are instead treated with one of the six FDA approved and better tolerated nucleos(t)ide analogs (NUCs) which inhibit reverse transcription by the multidomain HBV polymerase (P protein).

Early NUCs were prone to rapid viral resistance development, for lamivudine in up to 70% of patients after four years of treatment [4]. The current first-line NUCs entecavir, tenofovir disoproxil fumarate (TDF), and tenofovir alafenamide (TAF) are more potent and resistance rates are at most 1% after several years of treatment [5]. The high resistance barrier results from the massive suppression of viral genome replication, with >10^6^ fold declines in serum HBV levels, and the dependence on multiple mutations for the evolution of drug-resistant yet reasonably active enzyme P protein. Still, long-term (probably life-long) NUC therapy is necessary for most patients because, despite dampened liver inflammation, HBsAg seroclearance is rare. This is in part due to mRNA transcription from chromosomally integrated HBV DNA [6], a by-product of, but not essential for, the viral replication cycle, yet mainly to the NUCs not targeting the viral persistence reservoir, the covalently closed circular DNA (cccDNA) form of the viral genome [7]; cccDNA is produced in the host cell nucleus from the relaxed circular (rc) DNA present in infectious HB virions and is the template proper for transcription of the viral RNAs by host RNA polymerase II (see below). Hence, although formation of rcDNA containing nucleocapsids is largely blocked by NUCs, viral antigens, and immature RNA-containing nucleocapsids continue to be produced. Hence, upon therapy cessation virus replication can fully resume. As cccDNA may persist for decades, HBV reactivation can even occur long after a past self-limited acute hepatitis B [8] when immune control is lost by an unrelated disease or upon immunosuppressive treatment.

Global efforts have therefore been initiated towards new curative treatments for chronic hepatitis B, to achieve the sustained suppression of viral replication after treatment cessation, i.e., functional cure, and, ideally, complete elimination of all HBV genomes from the body [9], with several intermediate distinctions [10]. Various innovative therapeutic approaches are pursued [11] which can be divided into three main categories, namely, direct acting antivirals (DAAs) targeting viral factors [12,13], inhibitors of HBV-relevant host factors [14] including the entry receptor NTCP (see below), and immune activation, aiming to restore an adequate immune response against the virus, exhaustion of which is a hallmark of CHB [2,15,16,17]. Amongst the most advanced new DAAs are the capsid assembly modulators (CAMs), also called core protein allosteric modulators (CpAMs), capsid assembly effectors (CAEs), or, very simplifying, capsid inhibitors (CIs), which target the capsid-forming HBV core protein (Cp); they are in the focus of this review. Even though the phenotypic consequences of different CAMs may differ, their common mechanism of action (MoA) is to disable the proper dynamics of Cp and the capsid.

Excellent recent reviews on the general concept of therapeutically targeting viral structural proteins [18], analytical methods to monitor drug activities [19], and on specific CAM chemotypes [20] are available. Here we intend to provide a comprehensive and comprehensible overview on the functional dynamics of HBV Cp in the viral life-cycle, on the basics of the underlying biochemical and structural dynamics, and on up-to-date clinical data on the therapeutic use of CAMs against CHB.

## 2. Functional Dynamics of the HBV Core Protein and Capsid in Virus Replication

The notion of viral capsid proteins is largely shaped by the highly symmetric capsid structures they can form, classically determined by X-ray crystallography (see below). Capsids provide a virus with a stable container for its genome to travel through space and time, and many structural biology techniques rely as well on the stability of these particles. However, a look on the role of capsid proteins in viral replication in general, and that of the HBV Cp in particular, reveals a highly dynamic behavior which is crucial for maintaining the viral life cycle. 

### 2.1. Cp in Early Steps of the HBV Infection Cycle

**Figure 2:**
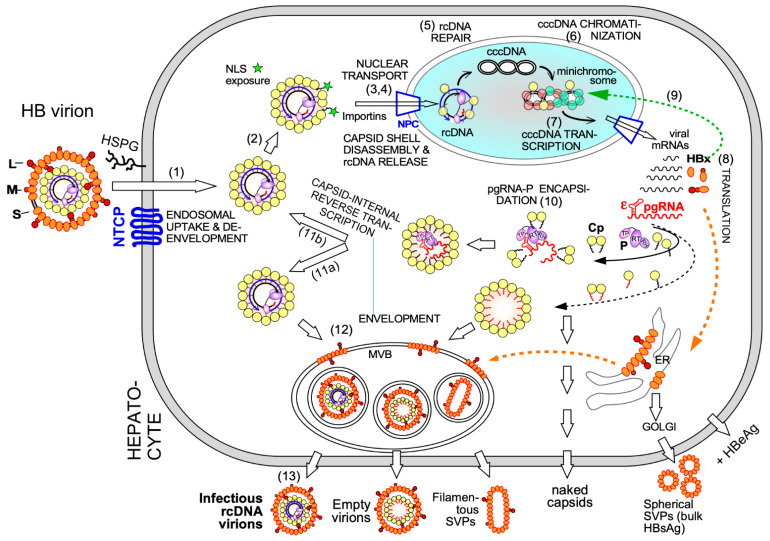
The HBV infectious cycle. The virion binds to heparan sulfate proteoglycans (HSPG) and via a high-affinity interaction of the L protein’s PreS1 domain to the hepatocyte-specific bile acid transporter NTCP, triggering entry into the cell. The subsequent steps leading to production of infectious progeny virions are numbered. (1) Endosomal uptake and loss of the envelope; (2) exposure of NLSs to importins; (3) nuclear transport and capsid shell disassembly at the nuclear pore complex (NPC); (4) release of the rcDNA genome into the nucleus; (5) repair to cccDNA; (6) minichromosome formation; (7) transcription by RNA polymerase II; (8) translation of viral proteins, including HBx; (9) stimulation of cccDNA transcription by HBx; (10) packaging of the pgRNA-P protein complex into newly forming nucleocapsids; (11) capsid-internal reverse transcription of pgRNA into new rcDNA; via (11a) to (12) envelopment of mature rcDNA containing nucleocapsids and (13) egress from the cell via the multivesicular body (MVB) compartment; or via (11b) recycling of rcDNA (2) to the nucleus to replenish the cccDNA pool. Infected cells also release empty virions, i.e., enveloped empty capsids and small amounts of enveloped RNA containing capsids (not shown). Further viral particles include non-enveloped “naked” capsids, and spherical and filamentous subviral particles (SVPs) comprising only envelope proteins. Secreted nonparticulate HBeAg arises from processing of the precore protein (not shown).

HBV infection begins with attachment of the envelope of rcDNA containing virions to cell surface heparan sulfate proteoglycans (Figure 2), including glypican 5 [21], and a high affinity interaction of the myristoylated N terminal PreS1 domain of the large envelope protein L (also termed large HBsAg) with a hepatocyte-specific bile acid transporter in the plasma membrane, sodium taurocholate cotransporting polypeptide NTCP, encoded by the SLC10A1 gene [22,23]. This interaction is largely responsible for HBV’s narrow host-range and strict liver tropism which have long hampered a better understanding of the early infection steps [24]. Fatty acylated PreS1-derived lipopeptides potently inhibit the interaction, and a specific myristoylated 47 amino acid peptide, Myrcludex B/Bulevirtide, marketed as Hepcludex, has been approved in the European Union as a first specific therapeutic against hepatitis D virus (HDV); this small RNA virus requires HBV’s envelope to produce infectious HDV particles and, thus, in patients is only found in coinfections with HBV [25,26]. Expectedly, Bulevirtide also suppresses HBV viremia but relapse after therapy cessation is common, owing to persisting cccDNA (see above). In contrast, HDV lacks a persistent form and a finite entry blockade can massively reduce and eventually eliminate HDV infection; similarly, chronic infections with hepatitis C virus (HCV), another RNA virus, can now be cured in most patients by finite DAA treatments [27]. 

At any rate does the HBV L protein–NTCP interaction trigger endocytic HBV uptake and, still poorly understood, loss of the envelope and release of the nucleocapsid into the cytoplasm. Here the dynamic nature of the nucleocapsid unfolds as previously hidden nuclear localization signals (NLSs) are exposed to the cellular karyopherins importin α and β which mediate transport, along microtubules [28], to the nuclear pore complex (NPC). There the capsid shell disintegrates and releases the rcDNA genome, which is covalently linked to the viral P protein, into the nucleoplasm [29]. In a multi-step process involving host DNA replication and repair factors [30,31,32] rcDNA is then converted into the plasmid-like episomal covalently closed circular (ccc) DNA form. 

Notably, Cp also contains nuclear export signals (NESs) [33], making it a nucleocytoplasmic shuttling protein [34]. Beyond previous evidence a recent study found Cp to shuttle, upon translation in the cytoplasm, with time and dependent on Cp concentration and assembly status, to the nucleoli, then to the nucleoplasm and eventually back to the cytoplasm [35]; more work will be needed to sort out the functional correlates of these different, apparently regulated, intracellular localizations.

### 2.2. Cp and HBV cccDNA

Chromatinization of cccDNA by cellular histone and non-histone proteins plus viral proteins enable its regulated use as transcription template for new 5′ capped and 3′ polyadenylated viral RNAs by cellular RNA polymerase II. Eliminating cccDNA would thus truly cure infection, a highly ambitious goal [36]. Easier to realize appears to silence transcription from cccDNA which depends on the viral HBx protein. A major function of HBx is to mediate ubiquitylation and subsequent proteasomal degradation of the cellular structural maintenance of chromosomes 5/6 complex (SMC5/6) which suppresses cccDNA transcription [37]. As part of this mechanism, or in addition, HBx seems to govern intranuclear cccDNA localization which in turn contributes to its transcriptional activity [38,39]. HBx and its interactions with cellular factors have thus also become new targets for therapeutic intervention [40,41].

Electron-microscopy [42] and, as for HBx [43], chromatin immunoprecipitation (ChIP) data suggest that also some Cp is associated with cccDNA, raising the possibility that Cp contributes as well to cccDNA stability and/or transcriptional activity. A nuclear Cp interactome study identified numerous cellular RNA binding proteins, amongst them serine- and arginine-rich splicing factor 10 (SRSF10) which suppresses HBV transcription [44]; the interaction with Cp may counteract such a restriction. Conversely, Cp has been proposed to recruit cellular restriction factors of the APOBEC family to cccDNA, inducing its (partial) degradation [45]; in this view, maintaining a long-term association of Cp with cccDNA [46] could even benefit APOBEC inducing antiviral approaches. More work will be required to firmly establish cccDNA regulatory activities for Cp; it should as well be considered that the rcDNA entering the nucleus comes associated with Cp, hence ChIP assays may detect residual Cp that escaped replacement by chromatin components. Along this line, recent studies have shown that a mutant HBV defective for Cp production (itself produced by trans-complementation with a Cp expression vector) is still infectious and able to generate transcriptionally active cccDNA [47] which remained stable for over two months [48]. This also argues that the intracellular recycling pathway (see Figure 2 step 11b), supposedly dependent on de novo Cp synthesis and highly active for DHBV [49,50], has only a minor role for HBV cccDNA replenishment, stability, or transcriptional activity, at least in this model. Blocking recycling might then not be very effective in reducing cccDNA levels. For DHBV the more active recycling and higher cccDNA copy numbers per cell may compensate the lack of a transcription-activating HBx-like gene product, possibly via extra functions of the larger avian HBV Cp [51]. Notably, other studies on HBV concluded that stable cccDNA levels are maintained by both genome recycling and de novo secondary infections [52]. Both mechanisms would support the rapid emergence of resistant viruses during therapy with early NUCs [4,53], either by adding the mutant cccDNA to the resident wild-type (wt) cccDNA in the nucleus, or by de novo deposition in naīve cells of only mutant cccDNA. Seemingly an academic issue, determining the relative contributions of the two cccDNA pathways is crucial for future therapy design [36]. If cccDNA is mostly replenished by de novo infection, entry blockers and, possibly, also CAMs targeting the incoming nucleocapsid could effectively drain the existing cccDNA pool but would be ineffective against intracellular cccDNA recycling. The reverse holds if recycling was the dominant pathway to cccDNA maintenance.

### 2.3. De novo Viral Protein Synthesis and Cp Functions in Progeny Virion Production

Transcription from cccDNA generates several subgenomic plus two greater-than-genome-length RNAs (Figure 1B). The subgenomic transcripts encode the large (L), middle (M), and small (S) envelope proteins and HBx. The shorter of the long transcripts, termed pregenomic (pg) RNA, has dual function as bicistronic mRNA for Cp and P, and as precursor for new rcDNA via capsid-internal reverse transcription. The 5′ terminally extended precore transcript is the mRNA for an N terminally extended version of Cp termed precore protein; as discussed below, the 29 aa extra sequence confers completely different properties to this protein [54,55].

The translation mechanism for the downstream P ORF on pgRNA is not clear but eventually one pgRNA molecule yields multiple copies of Cp per P protein. One of the next key steps for replication is co-packaging of pgRNA with P into newly forming nucleocapsids. This process is regulated on several levels, all aimed at coordinating capsid shell assembly with encapsidation of one molecule of P protein plus one molecule of pgRNA but no other viral or cellular nucleic acid; exclusively such nucleocapsids can give rise to progeny rcDNA and the next generation of infectious virions. Tellingly, in vitro reconstitution of this complex process has not yet been achieved although it would represent a more authentic model for studies on capsid-targeting drugs than the current in vitro systems (see below).

**Figure 3:**
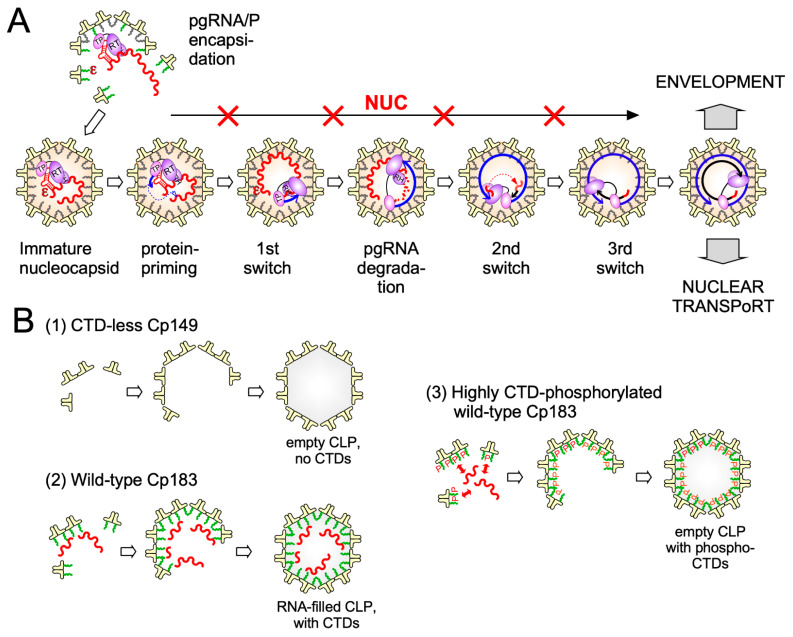
(**A**) The HBV capsid as distinct replication compartment. The pgRNA-P protein complex recruits Cp subunits, initiating assembly of nucleocapsids wherein pgRNA is reverse transcribed into rcDNA. P binding to ε also mediates protein-primed initiation of DNA synthesis at the ε bulge, covalently linking the TP domain to the DNA 5′ end. The short DNA oligo is transferred close to the pgRNA 3′ end (first switch) and extended towards the 5′ end, with degradation of the RNA in the nascent DNA-RNA hybrid. A 5′ terminal RNA oligo remains, is transferred to an acceptor not far from the 5′ end of the new minus-strand DNA (second switch), and primes plus-strand DNA synthesis. When reaching the template’s 5′ end a third switch to the 3′ end enables circularization and further extension into mature rcDNA. NUCs inhibit DNA synthesis but not immature nucleocapsid formation. Genome maturation is assumed to induce a structural change on the capsid that signals readiness for envelopment, and also for nuclear transport as part of the recycling pathway; in neither case is the new viral DNA exposed in the cytoplasm, minimizing immune recognition as non-self. Conceivably, the entire genome maturation process is accompanied by as yet unidentified conformational alterations in the capsid. RNA is shown as red wavy line, minus-strand DNA in blue, plus-strand DNA in black. Small amounts of ds linear (dsL) DNA from a failed second template switch are not indicated. (**B**) Nonproductive assembly paths of recombinant Cps expressed in *E. coli*. In the absence of the basic C terminal domain (CTD), Cps comprising an intact N-terminal assembly domain such as Cp149 assemble empty CLP shells, essentially via Cp dimer contacts; this is the subject of most current in vitro studies. In wild-type Cp183 electrostatic interactions between the R residues in the CTD and the sugar-phosphate backbone of bacterial RNAs contribute to assembly nucleation and stabilize the CLP against inter-CTD repulsion. In highly CTD-phosphorylated Cp183 the phosphoryl groups neutralize the R residues and block their general RNA binding capacity, yielding empty, phospho-CTD containing CLPs.

Clearly, however, the crucial initial event is specific binding of P protein to the 5′ proximal ε stem-loop structure (Figure 2 and Figure 3A) on pgRNA (though not the precore RNA; [56]), with the resulting ribonucleoprotein (RNP) complex nucleating capsid shell assembly [57] by a still ill-defined mechanism.

Notably, formation of genome-less capsid-like particles (CLPs) can also be initiated solely by protein-protein interactions and/or by non-specific protein-RNA interactions (Figure 3B; discussed in Section 4.3.4). A second key step triggered by the specific P-ε interaction is the protein-primed initiation of DNA synthesis on the internal bulge of ε by which the unique Terminal Protein (TP) domain of P becomes covalently linked to the 5′ terminal nucleotide of (−)-strand DNA, an evolutionarily ancient mechanism of genome copying [58]. In a complex process involving three template switches (Figure 3A) the pgRNA is eventually converted into partially double-stranded (ds) rcDNA [59]; as a byproduct some ds linear (dsL) DNA is formed, the predominant substrate for HBV DNA integration into the host chromosome which has no known function for virus replication but is a major source, beyond cccDNA, for HBsAg transcription [60]; this has also therapeutic implications [61,62]. 

The surface proteins S (small HBsAg, the major species) and its N terminally extended versions M (PreS2/S) and L (PreS1/PreS2/S) are synthesized at the endoplasmic reticulum (ER; see Figure 2); the vast majority of S is secreted as spherical capsid-less subviral particles (SVPs) with a low L content through the general secretory pathway [63]. The other morphotype are filamentous particles with a higher L content which are secreted through the multivesicular body (MVB) compartment [64]. Also complete enveloped virions egress from the cell via MVBs [65], dependent on the endosomal sorting complexes required for transport (ESCRT) machinery. Envelopment of matured nucleocapsids is mainly driven by interactions of the L protein, specifically a region near the C terminus of the PreS1 and the N terminus of the PreS2 domain (termed “matrix domain”), with still obscure signals on the capsid surface [66]. Capsid binding requires a cytoplasmic disposition of PreS1, equivalent to the virion interior, while cellular receptor recognition requires PreS1 on the virion surface. Both topologies are actually observed, likely controlled in temporal order [67]. Alternatively to nucleocapsid envelopment and secretion the newly produced nucleocapsids, like those from de novo infection, may recycle their rcDNA to the nucleus to enlarge, or replenish (if there is loss of) the cccDNA pool. Both pathways may be coupled to genome maturation (Figure 3A) which was long proposed to generate a conformational “maturation signal” on the capsid surface that indicates readiness for envelopment, based on data from DHBV [68]. Direct structural investigations have not yet detected marked differences between CLPs from wt versus mutant Cp like Cp F97L [69] which in cell culture shows a “premature secretion phenotype”, i.e., envelopment and secretion occur already with immature ssDNA containing nucleocapsids [70].

A further complication was the finding that most HBV capsids, including in infected patients, are devoid of nucleic acid yet still can be enveloped to give “empty virions” [71]; generation of empty capsids and virions in human cells is discussed in Section 3.3 and Section 3.4. The excess of enveloped empty and ds-DNA containing capsids over those carrying ss-DNA or RNA led to a “single-strand blocking model” whereby ss nucleic acids exert a negative signal for envelopment. Recent data indicate a more complex situation, with envelopment of empty vs. mature DNA containing capsids having both common and distinct determinants [72]. Using solid-state nuclear magnetic resonance (ssNMR; see below) we recently identified Triton X-100, used as a detergent during work-up of recombinant Cp capsids, as capable of specifically binding into a hydrophobic pocket in Cp and cause a major conformational shift in the capsid [73]. As many Cp mutations affecting capsid envelopment also contribute to this pocket, we proposed that occupancy of the pocket by a natural pocket factor, either cellular or viral, is involved in signaling readiness for envelopment. Realization of this concept will, however, require further experiments, as will the structural definition of the maturation dependent capsid features leading to recognition by the envelope proteins or their fueling into the recycling pathway.

Notably, also nonenveloped capsids can be released from cells [65,74,75]. In patients, such capsids may induce, and eventually be complexed by, anti-capsid antibodies which are detected in almost every HBV infection, regardless whether self-limited acute or chronic; the naked capsids, or a fraction thereof, may carry HBV-specific DNA and RNA species, including pgRNA [76,77]. Though many details need to be resolved, the overall picture indicates that also keeping viral and cellular components apart is an important function of the nucleocapsid. The RNA polymerase II transcribed HBV RNAs, including pgRNA, have the same features as cellular mRNAs, hence they are not recognized by innate sensors of nonself RNA (e.g., dsRNA or uncapped but 5′ triphosphorylated RNA) in the cytoplasm. Most positive-strand ssRNA viruses must actively prevent such recognition by physically separating their replication intermediates in virus-induced membraneous replication compartments [78]. However, the reverse transcription products of HBV pgRNA might be recognized as nonself DNA if freely floating in the cytoplasm, e.g., via the cyclic GMP-AMP synthase—Stimulator of Interferon Genes (cGAS-STING) axis [79,80]. Capsid-internal reverse transcription and controlled rcDNA release only at the nuclear pore prevent such encounters and contribute to the stealth virus nature of HBV [81,82].

## 3. Primary Structure and Biochemical Dynamics of HBV Core Proteins

Cp is the translation product of the C gene on pgRNA (Figure 1B), depending on the HBV genotype encompassing 183 to 185 amino acids; serologically it is defined as hepatitis B core antigen (HBcAg). No functionally relevant processing of Cp is known. In contrast, translation initiation at the preC AUG, present only on the 5′extended precore RNA (Figure 1B), yields the 29 aa longer precore protein which eventually ends up as secreted, non-assembled N- and C-terminally processed hepatitis B e antigen (HBeAg) discussed below. The ability to synthesize such precore proteins is conserved in most known animal HBVs yet during chronic HBV infection often precore-deficient variants are selected, resulting in the clinical condition of HBeAg-negative CHB; hence the precore protein and/or its HBeAg product are not essential for the viral life-cycle.

### 3.1. Domain Structure of HBV Cp

**Figure 4:**
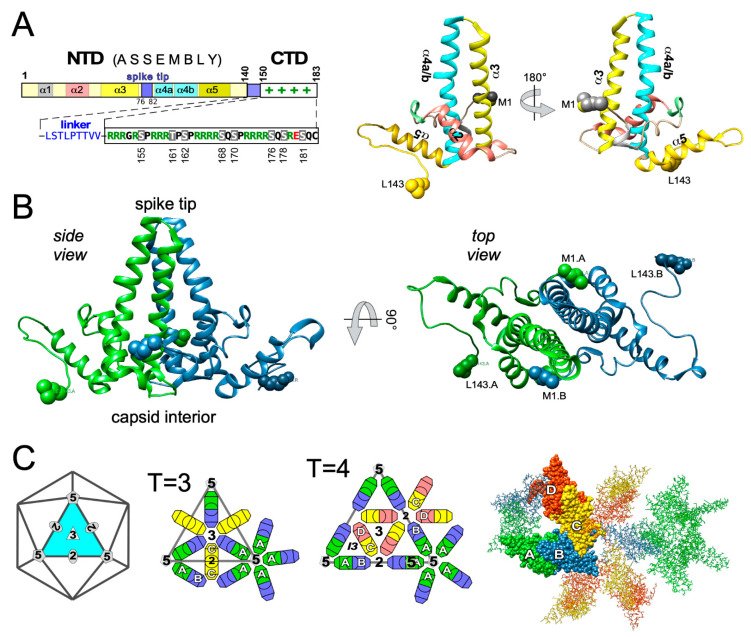
Basic structural features of HBV Cp. (**A**) Primary sequence and its correlation to the fold of the NTD. The bars symbolize the N terminal assembly domain (NTD), the linker (blue), and the basic nucleic acid binding C terminal domain (CTD). Numbers indicate amino acid positions, + signs the clustered R residues in the CTD. The linker and CTD sequences are explicitly shown. S and T residues in the CTD that are potential phosphorylation targets are highlighted; in recombinant Cp183 SRPK1 phosphorylates all but S181 and thereby blocks RNA binding. The models on the right (derived from PDB 6HTX) show two views of one monomer with the all-α-helical fold of the NTD. M1 and L143 are highlighted as spheres, the larger helices α2 to α5 are color-coded as in the linear representation on the left. The CTD is likely disordered and not resolved in current structures. (**B**) The Cp dimer. One monomer is shown in green, the other in blue. Dimerization relies on formation of a four-helix-bundle by the α3/α4 hairpins from two monomers, generating a prominent spike; in addition, the N terminal sequences embrace the spike base, as is most easily seen in the top view. (**C**) Cp dimer assembly into icosahedral capsid shells. On the left an icosahedron with its defining 2-fold, 3-fold, and 5-fold symmetry axes at one of the 20 triangular faces is shown. Cartoons in the center show the arrangement of Cp dimers in T = 3 and T = 4 particles. A subunits (green) cluster around 5-fold axes, B (blue) and C (yellow) subunits around 3-folds. The two C subunits are related by 2-fold symmetry and thus identical. In the larger T = 4 particle the two halves of the non-AB dimers are in distinct environments and thus termed C (yellow) and D (red). Counting the dimers per triangular face yields 4.5 for T = 3 and 6 for T = 4, hence totally 90 and 120 dimers, respectively. The model on the right shows the asymmetric unit, i.e., neighboring AB and CD dimers (residues as spheres), as seen in the context of a real T = 4 CLP (6HTX).

Engineered truncations of Cp expressed in heterologous systems, e.g., *E. coli*, indicated early on a two-domain structure (Figure 4A), in line with the unusual accumulation of clustered R residues downstream of V149 (16 R vs. 1 E). Shortening from the C terminus to residue 144 (Cp144) yielded soluble particulate protein while Cp138 was unstable and/or insoluble. Particles from Cps containing at least some of the C terminal arginines harbored easily detectable amounts of nuclease-protected bacterial RNA, those from Cp144 and Cp149 did not [83]. Hence Cp features an N terminal domain (NTD) required for assembly, and a highly basic CTD which binds nucleic acid, as confirmed for replicating virus in mammalian cells [84]. The sequence from position 140–149 is often regarded as linker but may well have more specific functions [85,86], including for the Cp phosphorylation status [87]. Owing to its much more efficient expression in *E. coli* compared to full-length Cp183 most structural studies were performed with CTD-less Cp149. Newer bacterial Cp expression vectors accounting for the different codon usage in *E. coli* vs. human cells, especially regarding the numerous R-codons in the CTD [88,89], now also allow efficient recombinant expression of Cp183, including in isotope labeled form enabling nuclear magnetic resonance (NMR) studies [90,91]. More detailed three-dimensional (3D) features of Cp are discussed in Section 4.

### 3.2. HBV Precore Protein—A Nonassembling Secretory Cp Derivative

**Figure 5:**
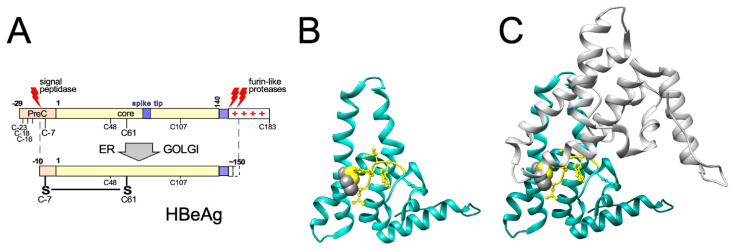
HBeAg, a distinct structure despite nearly identical primary sequence to the Cp assembly domain. (**A**) Processing pathway of the precore protein. Cotranslation of the 29 codons of the preC region with the core ORF generates the precore protein. The first 19 PreC aa act as signal peptide directing the protein into the ER where N terminal cleavage occurs; during transit through the secretory pathway furin-like proteases clip-off most of the basic CTD, and in the oxidizing environment a specific disulfide bridge is formed between C-7 and C61 which determines the distinct structure of HBeAg. Failed signal peptide cleavage can create an only C terminally processed recently termed PreC protein (not shown) that contributes serologically to the “hepatitis B core-related antigen” (HBcrAg). (**B**) Crystal structure of one monomer in the HBeAg dimer (PDB 3V6Z). The preC-derived extra sequence is shown in yellow; C-7 and C61 are depicted as spheres. The second monomer is omitted for clarity. (**C**) The HBeAg dimer. The second monomer is shown in grey. Note the completely different dimerization mode compared to Cp which creates new epitopes, exposes epitopes that are hidden in the assembled capsid, and prevents HBeAg multimerization.

The precore protein undergoes several processing steps before it is found in the circulation as HBeAg [92]. It comprises the entire information of Cp, preceded by the 29 aa PreC sequence which determines its distinct antigenic and structural properties (Figure 5A). The first 19 aa act as a cleavable signal peptide (“presequence”) that directs the protein into the cell’s secretory pathway; during further transit, furin-like proteases clip-off the CTD. Eventually HBeAg is identical to Cp except it carries a 10 aa N-terminal extension (“prosequence”) and lacks about 30 aa from the C terminus [93]. Failed N terminal processing yields a 22 kDa precore protein, renamed as PreC which together with HBeAg and HBcAg constitutes the “hepatitis B core-related antigen” (HBcrAg) that has gained interest as secreted biomarker for cccDNA transcriptional activity, although with some dissent [94]. While not essential, the precore-derived proteins may have immunomodulatory and tolerogenic properties, e.g., by affecting interleukin expression [95], that favor chronicity of infection [96].

Biochemically most interesting are the structural consequences of the prosequence which contains one of the four cysteines in the preC region [97]. This cysteine (C-7) forms a specific intramolecular disulfide bond with C61 [55] which completely changes the way in which HBeAg dimerizes [54] (Figure 5B,C) compared to Cp (see Section 4.2) and prevents it from assembling into particles although the entire assembly domain of Cp is present.

### 3.3. Posttranslational Modifications of Cp

The multiple and in part opposing functions of Cp in the viral life-cycle, such as capsid assembly vs. disintegration, nucleic acid packaging vs. release, or nuclear transport vs. export, imply a tight regulation for which posttranslational modifications (PTMs) seem to be crucial. Several such PTMs have been reported, including dimethylation of Arg residues by protein arginine methyltransferase 5 (PRMT5) and ubiquitylation [98,99]. Notably, transient regulatory modifications such as with small ubiquitin-like modifier (SUMO) might occur at few subunits, be difficult to detect, and still affect the entire capsid. However, the exact functions of these PTMs remain to be established.

The single-most important Cp PTM is phosphorylation, first indirectly suggested by an “endogenous kinase activity” associated with HBV capsids purified from serum virus [100,101] and directly seen for DHBV Cp where, different from HBV Cp, phosphorylation visibly affects the protein’s mobility in one-dimensional SDS-PAGE [102]. Such data suggested dynamic changes in the DHBV Cp phosphorylation status during the life-cycle, with a high level in intracellular capsids and little or no phosphorylation in Cp from mature rcDNA-containing DHB virions in serum, as confirmed by mass spectrometry [103]. Mutagenesis studies replacing hydroxy amino acids in HBV Cp, many in the CTD (Figure 4A), provided some insights but interpretation suffered from the unknown degree of phosphorylation per Cp molecule, and in particular from the static nature of amino acid replacements. For instance, replacing most if not all potential phosphorylation sites by alanines, as a mimic of non-phosphorylated Cp, completely blocked capsid-internal reverse transcription, suggesting phosphorylation is crucial for HBV replication. Phosphorylation-mimicking exchanges of serine to acidic amino acids allowed ssDNA but not rcDNA or dsL-DNA formation [104,105]. Related studies led to a “charge-balance” model [106,107] whereby the replicative functions of the nucleocapsid depend on electrostatic homeostasis between the positive charges of basic residues in Cp, mostly the CTD, and the negative charges of the packaged nucleic acid at the respective stages of genome maturation. In the simplest view, the Cp CTD provides 15 net positive charges (16 R vs. 1 E residue), and most capsids comprise 240 Cp subunits (see below). The resulting total of 3600 positive charges would then be able to “neutralize” roughly as many negative charges from nucleic acid; several studies concluded that recombinant Cp183 capsids package the equivalent of ~4000 nt bacterial RNA [89,107,108,109]. Assuming 1.4–1.6 RNA phosphates per Arg residue [110] this would translate into a total binding capacity of around 5500 nt, equivalent to rcDNA with a complete 3200 nt (−)-strand DNA and a two-thirds completed (+)-strand, as often observed for HBV rcDNA from virions [111]. In line with the importance of charge homeostasis, nucleocapsids from Cp variants with fewer positive charges in the CTD, e.g., by CTD truncation, exchange of R residues or introduction of acidic side chains, commonly become unstable upon formation of mature ds-DNA; instead nucleocapsids containing shorter ds-DNAs derived from encapsidated spliced pgRNAs (which seem not essential for viral replication) accumulate [107,112,113].

### 3.4. Dynamic Cp Phosphorylation/Dephosphorylation for Regulated Nucleic Acid Binding

**Figure 6:**
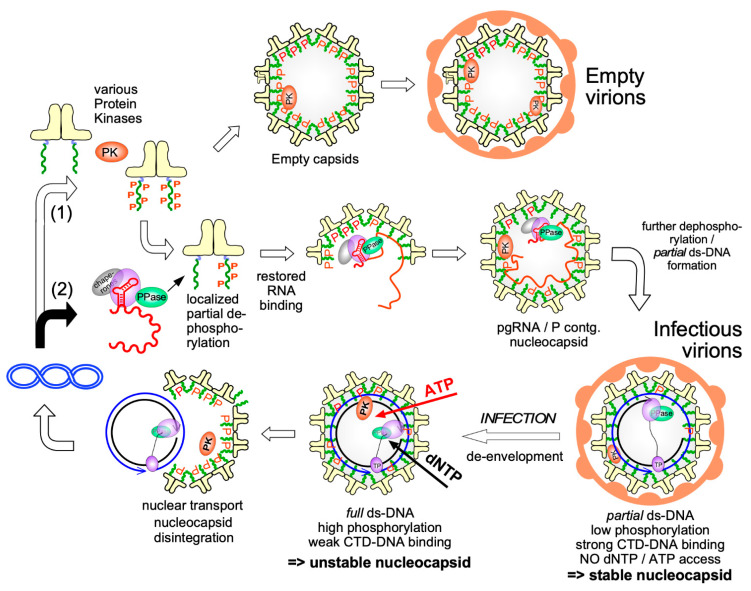
A model correlating HBV Cp phosphorylation and replication dynamics. By default, Cp produced in hepatocytes (1) is a substrate for various protein kinases (PKs), causing high-level phosphorylation, suppression of nucleic binding capacity and eventually empty virion formation; this prevents packaging of non-relevant viral and cellular RNAs. For infectious virion production (2) the model predicts that specific encapsidation of the pgRNA-P complex is enabled by the localized dephosphorylation of nearby Cp dimers by (a) protein phosphatase(s) associated with that complex. This restores the previously suppressed RNA binding capacity of Cp, with dephosphorylation of further Cp subunits enabling interactions that result in progressive and, eventually, complete packaging of pgRNA into newly forming nucleocapsids, and, subsequently, infectious virions in which Cp is largely unphosphorylated. The envelope blocks access of ATP and dNTPs. Upon infection of a new cell, removal of the envelope (de-envelopment) allows influx of dNTPs and ATP into the particle, enabling further fill-in of the plus-strand DNA, and re-phosphorylation of previously dephosphorylated Cp subunits. In effect, this increases conformational stress from the increasing DNA stiffness, and reduces Cp’s nucleic acid binding capacity, weakening the nucleocapsid structure. Eventually, this destabilization mediates disintegration of the capsid shell and release of the rcDNA into the nucleus to start of a new cycle.

As phosphorylation reduces the positive charge per Cp and hence the capsid’s nucleic acid binding capacity the assumed dependence of HBV pgRNA packaging and replication on phosphorylation is not obvious. In particular, given the hundreds of protein kinases in mammalian cells [114], the number of potential phosphorylation sites in Cp and their combinations is enormous, explaining discrepant proposals on which kinases are relevant for Cp. In a reductionist approach we recently established a bacterial co-expression system for Cp plus select mammalian protein kinases which exploits the fact that bacterial kinases do not phosphorylate Cp at all. Applied to serine-arginine-rich protein kinase 1 (SRPK1), one of the assumed Cp kinases [115] and combined with Phos-Tag SDS-PAGE [116], mutagenesis and mass spectrometry we identified seven of the eight hydroxy amino acids in the CTD (all but S181) as SRPK1 substrates and showed that this level of phosphorylation suppresses bacterial RNA packaging as efficiently as deleting the entire CTD (Figure 3B). In mammalian cells most Cp was found to be as highly or even higher phosphorylated [89], perhaps at NTD-borne residues, such as S44 and S49 [117]. In line with efficient suppression of general RNA binding most capsids in human cells have been found empty [118]. Hence high-level phosphorylation is indeed important for HBV replication as it prevents non-specific RNA packaging. At the same time this raises the question as to how specific packaging of the pgRNA-P protein complex can proceed (Figure 3A). Even if the initial nucleation event might rely on non-electrostatic interactions, e.g., between P protein and Cp, eventually the entire pgRNA must be accommodated inside the capsid shell. We therefore proposed that pgRNA-P encapsidation goes along with dephosphorylation of the participating Cp subunits but not the bulk of Cp in the cell. Temporal and spatial coupling could be achieved by a protein-phosphatase activity that is associated with the pgRNA-P protein complex. Several recent studies support this concept [119]. Protein phosphatase 1 (PP1) was found packaged in pgRNA carrying nucleocapsids but not in empty capsids [120]. PP2A, a regulatory subunit of which has binding motifs in the Cp linker, may lateron play a role in dephosphorylation [87]. New data also further support a role of cyclin-dependent kinase 2 (CDK2) in Cp phosphorylation, as part of the endogenous kinase activity [85]; notably the presence of CDK2 is not dependent on pgRNA or P protein, indicating its recruitment by Cp. These data are combined in a working model for phosphorylation/dephosphorylation controlled specific pgRNA-P protein packaging and reverse transcription (Figure 6). In essence, Cp phosphorylation would occur by default through (a) Cp-associated kinase(s) and eventually yield empty virions. Dephosphorylation would be triggered by (a) phosphatase(s) associated with the pgRNA-P protein RNP. In line with electrostatic homeostasis, dephosphorylation would continue during packaging of the entire pgRNA and its reverse transcription into rcDNA until all CTDs are unphosphorylated, yielding infectious virions. Upon infection, the influx of dNTPs and ATP would enable DNA completion and re-phosphorylation, destabilizing the nucleocapsid and preparing it for rcDNA release at the nuclear pore.

## 4. Overall Structural Dynamics of HBV Cp

The most evident property of Cp is its ability to self-assemble into icosahedral capsids, even without additional viral or mammalian cellular factors. In this section, we focus on high-resolution structural analyses of the capsid and Cp, and on the assembly pathway. 

### 4.1. Early Evidence for Autonomous Cp Self-Assembly into Capsid-Like Particles

Monitoring structural dynamics requires a defined reference structure. Early studies enriched bacterially expressed Cp as CLPs by sedimentation velocity and/or size exclusion chromatography (SEC). Negative stain transmission electron microscopy (TEM) and their distinct mobility in native agarose gel electrophoresis (NAGE) distinguished them from nonspecific aggregates. NAGE separates intact capsids based on surface charge and, depending on the gel’s pore size, on particle size; only homogeneous particles appear as distinct bands. Using nucleic acid stains, e.g., ethidium bromide, the presence of nucleic acids in the particles can easily be monitored [83] which revealed the importance of the CTD for general RNA packaging. Oxidative cysteine–cysteine crosslinking was consistent with a network of multiple dimers in the structure [121]. Cp mRNA injection into *Xenopus* oocytes also indicated formation of a pool of Cp dimers which rapidly assembled into icosahedral capsids, without tangible intermediates [122]; the cooperative process required, in this system, a critical concentration of ~1 µM Cp [123].

A breakthrough in the structure determination of HBV CLPs and the Cp subunit came with cryogenic EM (cryo-EM) where the biological specimen is frozen in vitreous (i.e., amorphous) ice in a hydrated near-native state, rather than dehydrated and stained with a heavy metal salt in negative staining. Such data revealed for recombinant CLPs a dimorphism of 90 Cp dimer and 120 Cp dimer particles [124] in icosahedral T = 3 and T = 4 arrangements, with diameters of about 32 and 36 nm, respectively. 

#### T = 3 vs. T = 4 Dimorphism of HBV Cp Capsids

Based on the concept of genetic economy, Crick and Watson predicted [125] virus capsids to consist of multiple copies of one or few capsid proteins. Having the same structures their interaction interfaces would also be the same, hence the assembled capsids would be symmetrical, either helical (filamentous viruses), or icosahedral (spherical viruses). The icosahedron is the largest of the Platonic solids, built from 20 equilateral triangles forming the 20 faces, 12 vertices at the five-fold and 30 edges at the two-fold symmetry axes (Figure 4C). While proteins are intrinsically asymmetric icosahedral symmetry can be achieved by symmetrically arranging three subunits on each of the 20 faces; hence 60 identical subunits employing identical interactions to their neighbors form the simplest icosahedral virus capsids, e.g., for the tiny satellite of tobacco necrosis virus (STNV). Their limited nucleic acid packaging capacity led Caspar and Klug to formulate the quasi-equivalence principle [126] whereby the subunits are allowed to adopt a number of similar but not strictly identical (“quasi-equivalent”) conformations. Hence, they can stably exist in slightly different environments, most notably as pentamers around the five-fold axes, and hexamers around the three-fold axes. Geometrically this can be envisaged as subtriangulation of the 20 icosahedral faces, with allowed triangulation numbers (T) given by the equation T = h^2^ + hk + k^2^ where h ≥ 1 and k ≥ 0. The simplest 60 subunit case is defined by h = 1 and k = 0, hence T = 1. The next allowed numbers are then T = 3 (h = 1, k = 1) and T = 4 (h = 2, k = 0). Figure 4C shows schematically the respective subtriangulations into 3 and 4 facets per face creating 3 and 4, respectively, distinct environments and subunit conformations. Analogously, the T number indicates the multiples of 60 that make up the total number of subunits in that particle, which is always composed of 12 pentamers and 10(T-1) hexamers. Further information can be found in a recent historic review [127] and on the educational portal of the Protein Data Bank under “https://pdb101.rcsb.org/learn/flyers-posters-and-other-resources/poster/200-icosahedral-viruses (accessed on 20 October 2021)”.

For the HBV capsid, T = 3 and T = 4 symmetry translate into 180 and 240 Cp monomers per particle, yet owing to the stable association of two monomers the particles are more appropriately described as composed of 90 and 120, respectively, clustered dimers (Figure 4C). The corresponding subunit conformers are conventionally termed A, B, and C for T = 3 symmetry, and A, B, C, and D for T = 4 symmetry; in the clustered dimer HBV capsid structures this is realized as AB and CC dimers in T = 3, and AB plus CD dimers in T = 4 capsids. The A subunits are arranged around the five-fold axes, all others are part of the different hexameric environments. The most obvious manifestation of quasi-equivalence is that the same interaction region in Cp can mediate stable association with four (at the five-fold) or five partners in both T = 3 and T = 4 particles. The biological background for the dimorphism of the HBV capsid is unclear. Both morphotypes were also found for capsids released from serum HBV [128,129] and analyzed as native enveloped particles [130] but the larger T = 4 particles dominate by far. Recombinant CLPs from most Cps also assemble to >90% into T = 4 particles, except for heavily truncated derivatives such as Cp140. However, both forms can bind CAMs [131] and the presence of CAMs during assembly could affect their ratio as the particle types display subtle but distinct differences in subunit conformation and stability [132].

### 4.2. High-Resolution Structure Determination of HBV Capsids

**Figure 7:**
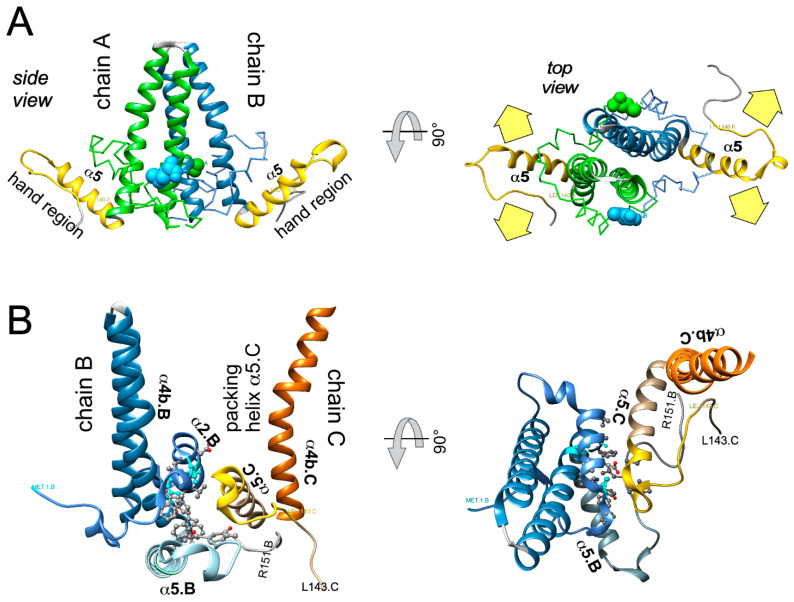
The HBV Cp inter-dimer interaction. (**A**) The Cp dimer is tetravalent. The “hand region” comprising helix α5, the P-rich turn and the downstream sequence to the end of the assembly domain at L140 (highlighted in gold for each monomer) is the main module for inter-dimer contacts. Each hand region provides two interfaces, hence the dimer is tetravalent, as indicated by the broad yellow arrows in the top view. Only helices α3, α4, and α5 are shown as ribbons. (**B**) Close-up of the B–C interdimer contact. For chain B the entire NTD is represented, for chain C only the descending helix α4 and the hand region. Properly oriented chain B residues from F23 through helix α2 (P25 to Y38), from α4b (W102 to F110), plus W125 and residues from P138 to S141 form a largely hydrophobic cavity, against which α5 from chain C can snuggly pack, in particular via V120, V124, W125, R127, T128, Y132, R133, P134, and P138. In the capsid, the respective residues undergo analogous interaction with their other neighbors. The pocket formed by the dimer–dimer interaction is the target for all currently known CAMs (“HAP pocket”) and the main site for CAM resistance confering mutations.

Cryo-EM plus image reconstruction together with existing biochemical data enabled to solve the structure of the Cp assembly domain at ~7Å resolution [133,134], revealing a largely α-helical fold (Figure 4A). The central two of the five α-helices (α3 and the bipartite α4, with α4a and α4b) from two adjacent Cp monomers associate into a four-helix-bundle that forms a spike protruding from the capsid surface (Figure 4B). Helices α1 and α2 embrace the bottom of the spike. Interdimer interactions rely mainly on helix α5 from R112 (after the hinge residue G111 following α4b) to T128 and its downstream sequence to at least position 140 [133] which folds back on α5 through a proline-rich turn including P129/P130 and P134/P135; the entire subdomain is also termed “hand region” [135]. This explains why truncations upstream of L140 rendered Cp assembly-defective [83,136]. Further detail on the inter-dimer contacts became visible, at 3.5Å resolution, in the first X-ray crystallographic structure of Cp149 CLPs [137]; the respective Protein Data Bank (PDB) entry 1QGT is widely used as a reference structure. Each of the two hand regions per dimer has two interaction sites (Figure 7A), making the dimer tetravalent. As shown in Figure 7B for the B-C contact, residues from α2, α4b, and the hand region from chain B form a largely hydrophobic cavity which can snuggly accommodate residues from α5 of the neighboring C chain (the “packing helix”). This pattern is reiterated at all dimer–dimer contacts in the particle. Notably, the narrow cleft between the interacting dimers comprises the binding pocket for all currently known capsid assembly modulators (see Section 5.3).

#### Crystal-Independent High-Resolution Cp Analysis

While X-ray studies can achieve resolutions down to below 1.5Å, the length of a covalent C-C bond, a major drawback is the need for crystallized samples which, in turn, is hampered by sample heterogeneity, including T = 3 vs. T = 4 dimorphism (see above). In cryo-EM, by contrast, single particles on the micrographs can be selected for image reconstruction. Averaging thousands of such particles then yields higher-resolution information, initially down to about 4Å [138], where the protein backbone and bulky sidechains became visible. The subsequent “resolution revolution” [139] brought by new detectors pushed this limit to below 3Å which reveals many, including smaller, sidechains. The currently highest resolution cryo-EM derived model for HBV capsids from full-length Cp183 (PDB 6HTX) has a nominal resolution of 2.66Å [69] but even higher resolution is principally possible [140]. Hence cryo-EM and cryo-electron tomography (cryo-ET), which generates multiple views of the same particles from different angles by specimen tilting, have become a mainstay for the study of virus structure [141]. 

Structural information on the Cp CTD is scarce as most studies used CLPs from CTD-less Cp; yet also in recent structures such as PDB 6HTX only a few residues downstream of the assembly domain are sufficiently ordered to be visible. In line with earlier findings [142] the CTD residues are located inside the particle although for nuclear transport via importins one or more CTDs must be extruded through the capsid shell [143]. Likely such extrusion is only possible when the internal interaction between CTDs and packaged nucleic acids is weakened, such as by phosphorylation (see Figure 6).

Remarkably, the HBV Cp NTD fold is also found in Cps of ancient nonenveloped fish viruses, the nackednaviruses, distant relatives of HBV in terms of genome organization, replication mechanism [58] and Cp size but not primary sequence [144]. In contrast, avian HBVs and some amphibian and reptile HBVs employ larger Cps of ~260 aa in length. A recent 3.5Å cryo-EM structure of CLPs from the full-length DHBV Cp [51] revealed a similar dimeric α-helical fold in the capsid shell region but major differences in the upper half of the spikes and the N terminus whose position in HBV Cp is taken over by C terminal residues in DHBV Cp. Most striking is the presence of a central 45 aa proline-rich, slowly folding extension domain that leans on and almost covers the spike tips when folded and thus affects the entire capsid surface. Likely the large Cps represent a distinct evolutionary adaptation to the dynamic requirements of the hepadnaviral lifecycle; elucidation the underlying mechanism would certainly also benefit a deeper understanding of HBV. 

Underlining the high stability of the HBV Cp fold and the capsid structure, heterologous sequences can be fused to the Cp sequence without inhibiting assembly. N terminal fusions locate to the CLP exterior [145], C terminal fusion to Cp149 of a 17 kDa nuclease gave intact particles carrying functional enzyme domains in their lumen [146], in line with internal localization of the Cp CTD. The strongest exposure, commonly desired for particulate presentation of antigens in vaccinology, was, however, achieved by inserting heterologous sequences, including natively folded green fluorescent protein (GFP), into the small loop at the spike tip [147]; notably, such Cp-GFP fusions assembled also in mammalian cells but supported HBV replication only as mosaic particles with wt Cp subunits (P. Kratz and M. Nassal, unpublished observations). Insertion of unfavorably shaped proteins such as the extended outer surface protein A (OspA) from *Borrelia burgdorferi* interfered with particle assembly. However, splitting the fusion protein chain upstream or downstream the insert, thus fixing the foreign sequence over one end only, yielded soluble CLPs [88,148]. Also split Cp on its own was efficiently expressed in bacteria, corroborating stability of the NTD fold. Beyond applied aspects spike insertions enabled to probe the conformational flexibility of the capsid itself. Cryo-EM reconstructions of GFP presenting non-split CLPs with progressively shortened linkers between insert and helices α3 and α4a revealed significant intra-subunit as well as inter-subunit movements compared to wt CLPs, with the helices moving as rigid rods connected by pivots such as G111 between helices α4b and α5 [149]. Hence as in a mechanical linkage of bars structural changes can propagate through the subunits and the entire particle. In line with recent dynamics data [150,151] iterative back-and-forth movements might be important during the capsid-internal DNA polymerization steps.

**Figure 8:**
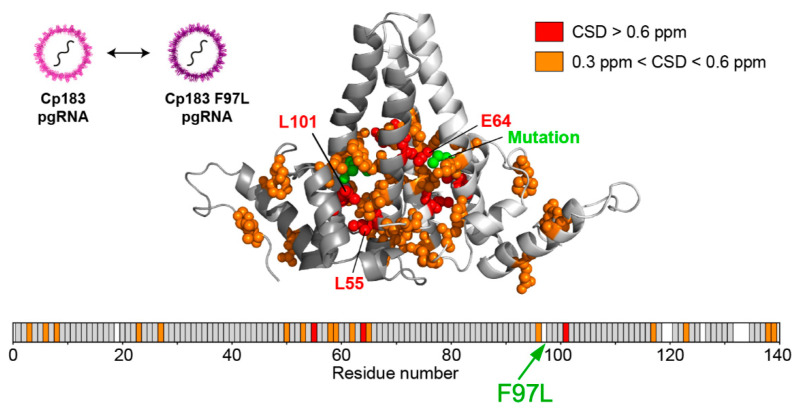
Solid-state NMR can detect conformational alterations with high resolution at near-native conditions. Once all resonances are assigned in a reference structure other, similar samples can be compared against the reference. In the example recombinant pgRNA loaded CLPs from wild-type Cp183 and its variant F97L were compared; in cell culture this mutation relaxes the restriction on envelopment of nucleocapsid carrying immature genomes (adapted from Lecoq et al.). Residues whose conformational environment changes by the mutation experience chemical shift differences (CSDs) compared to the reference; larger differences indicate larger alterations. The linear representation at the bottom assigns such residues to the primary sequence, the model above to the structure of the Cp dimer. Note that numerous residues not only in immediate vicinity to the mutation are affected. The impact of bound ligands on the structure can similarly be determined, even in irregularly assembled multimers. Figure courtesy of Lauriane Lecoq and Anja Böckmann.

High-resolution structural analyses of more subtle Cp mutations can also provide information on capsid-intrinsic conformational plasticity, e.g., changes induced by the F97L mutation in a hydrophobic pocket formed at the intra-dimer interface [69]. Notably, solid-state nuclear magnetic resonance (ssNMR) is gaining increasing interest as high-resolution structural technique because it can be performed under near-physiological conditions, including temperature, and because it can detect conformational alterations at individual protein residues with atomic resolution [152]. As the characteristic NMR chemical shifts are influenced by the environment comparison with a reference structure of capsids from wt vs. mutant Cp, or similarly of neat capsids vs. capsids with a bound ligand [73], can thus accurately reveal where changes happen. Examples include the hinge residues in Cp [91] that distinguish the four quasi-equivalent A, B, C, and D conformers in a T = 4 particle (Figure 4C); alterations caused by mutations, e.g., the F97L exchange (Figure 8); or changes induced by capsid-binding heterologous molecules such as the detergent Triton X100 [73] yet also therapeutically relevant molecules. Recent technical progress resulted in ever lower sample size requirements (in the submilligram range), and higher magnetic fields in proton resonance frequencies of 1200 MHz in ever higher resolution [153]. 

### 4.3. Monitoring Assembly Dynamics

A most fundamental issue for understanding capsid proteins is to define the transition from individual subunits to the assembled particle, including potential intermediates, and vice versa disassembly. Static methods, such as X-ray crystallography or cryo-EM, may yield important indirect clues but rarely allow in situ monitoring. However, several newer, including high-resolution and single-molecule techniques are now available which we will very briefly touch upon. For a detailed and comprehensive description readers are referred to an excellent review [19].

#### 4.3.1. Disassembly–Reassembly Studies

Crucial for all such studies is access to the capsid protein in either the non-assembled or the assembled state. A straightforward approach is to look at the end products of Cp translation in a given expression system. Obviously, the initial products must be individual Cp protein chains which then may associate into the final capsid or CLP structure. More control over the assembly conditions and the impact of individual “co-factors” came from disassembly/reassembly systems. Bacterially produced CLPs from Cp149 and other CTD-less Cps can be dissociated into stable dimers by mildly denaturing conditions, e.g., 1.5–3 M urea at moderately alkaline pH and low ionic strengths. Isolated dimers can then be reassembled by lowering the pH and denaturant concentration, and by increasing ionic strength [154]; hence the denaturing conditions were sufficiently strong to break the interdimer contacts but maintained the fold of the dimeric subunits in an assembly-competent state. Similar protocols also enabled co-assembly of different Cp variants and/or Cp carrying different fluorescence labels, e.g., for Fluorescence Resonance Energy Transfer (FRET) experiments [155,156]. One of the general conclusions was that with -3 kcal/Mol the free energy gain per dimer–dimer contact is rather low under physiological conditions, and stability of the particle as a whole depends on the multitude of these contacts [157]. Biologically, such weak interdimer interactions allow for proof-reading of aberrantly assembled particles because improperly incorporated subunits can be released and reintegrated [158].

By contrast, disassembly of CLPs from CTD-carrying Cp with packaged RNA required harsher conditions, indicating stabilizing effects of additional RNA-Cp interactions; along this line, the empty CLPs from high-level phosphorylated Cp183 are as easily disassembled as those from CTD-less Cp variants [89]. Only in 2010 were proper disassembly conditions for RNA-containing CLPs from Cp183 established that maintained the subunits’ reassembly competence [108]. This allowed investigations on the impact of cargo, CTD length, and chemical modifications on assembly efficiency. Moreover, advanced techniques now yield information not only on whether or not assembly can occur at all but also on how it proceeds.

#### 4.3.2. Specialized Techniques to Study Cp Assembly

Measuring assembly pathways and rates, including under different conditions and/or the presence of molecules affecting the rate, requires techniques monitoring the temporal changes in the proportions of unassembled precursors vs. end products or possibly tangible intermediates.

X-ray crystallography and cryo-EM rely on sampling many identical particles; heterogeneity in the specimen will eventually cause a loss of resolution. NMR can monitor changes in the vicinity of NMR-active nuclei over a broad range of timescales [152] but is also an “ensemble technique” that reports on the average properties of a population of molecules or particles. Various less commonly known techniques are highly useful, especially in combination, to follow assembly some of which we will briefly address; for more information see reference [19]. 

A basic optical technique is light scattering which increases with particle size. Several variations exist, recently also for single-particle imaging, similar to fluorescence correlation spectroscopy. Mass spectrometry (MS) can also offer single-particle resolution, and especially charge-detection MS (CDMS) has been used to identify numerous small transitory as well as more stable “late-stage” Cp assembly intermediates, with masses between those of T = 3 and T = 4 HBV capsids [159]. Another advance are microfluidic and nanopore-based techniques, such as resistive-pulse sensing where particles of different sizes passing through the pore cause correlating changes in conductivity. Atomic force microscopy (AFM) can provide additional information on the mechanical properties of particles, including how they are affected by the material inside the particle, e.g., nucleic acid. Not the least, theoretical approaches such as molecular dynamics (MD) simulations can now handle an entire capsid [160]. A recent all-atom MD simulation of HBV capsids bound with a CAM compound suggested a mechanism for crosstalk between intra- and inter-dimer interfaces and thus a way how allostery can traverse through the entire Cp dimer [161] that is in line with experimental data [162]. Another finding was a population of dimers with significantly splayed intra-dimer interface; such opening of the four-helix bundle has also been seen in experimental studies, e.g., when capsids interact with spike-binding peptides [163], or by mutation of the spike tip-located D78 residue which, again, affects the rate of assembly [151]. While the biological meaning is not yet resolved, these convergent data exemplify how different approaches can eventually point into the same direction. Combining the different techniques can therefore provide unprecedented new insights into HBV capsid assembly as well as its inhibition; for instance, owing to the crosstalk throughout the dimer, compounds acting on the intradimer interface or the spike tip could similarly act as allosteric assembly modulators as the currently dominating CAMs that target the interdimer interface.

#### 4.3.3. Cp Assembly Intermediates

While new technologies promise much more detailed insights into HBV capsid assembly some basic aspects of the pathway are already established. Assembly proceeds most likely by a nucleation mechanism (comparable to the induction of crystallization from an oversaturated solution when a “crystal seed” is added), i.e., the rate-limiting assembly step is the formation of an intermediate (the nucleation seed) to which additional dimeric subunits can be added (see Figure 3), in the elongation phase, on a downhill energetic path [164]. The likely nucleation seed for neat CTD-less Cp derivatives such as Cp149 is a three-fold symmetric trimer of dimers [165], as supported by recent EM evidence [132]; this study identified, in addition, a two-fold symmetric pentamer as another predominant intermediate; there, two more dimers are associated to a trimer of dimers so as to generate two adjacent triangles. Considering that each Cp dimer is tetravalent (Figure 7) the trigonal trimer of dimers saturates 6 of the 12 valencies, the two-fold symmetric pentamer 12 of the totally 20 valencies; hence these arrangements minimize the number of free valencies. As assembly proceeds, the percentage of free valencies per subunit decreases further until all valencies are saturated upon addition of the last dimer. In turn, removing the first subunit from a fully assembled capsid has the highest energy cost [18], hence assembly and disassembly can be nonsymmetrical processes showing hysteresis [166]; sophisticated techniques such as time-resolved small angle X-ray scattering confirm this view [150]. Moreover, assembly may proceed via more than one pathway, as indicated by the observation with single-particle techniques such as CDMS of particles with fewer (“defective”) or more subunits (“overgrown”) than those defining a perfect icosahedron. Both defects can slowly be corrected, suggesting that completion is a separate phase in assembly [158]. Also a recent single particle cryo-EM study found defective particles to account for a substantial fraction of the total population, perhaps reflecting that nature can live with, or even exploits, imperfection in capsid assembly [167]. The actual assembly conditions are also important for the pathways; mild conditions (including low Cp concentration and ionic strength) favor regular T = 4 CLP formation with few tangible intermediates, aggressive conditions (including high Cp concentration and ionic strength) favor formation of larger, kinetically trapped intermediates and a higher proportion of T = 3 capsids. However, the correlation of any of these in vitro conditions with assembly of replication-competent HBV nucleocapsids remains to be examined.

#### 4.3.4. Specific Versus Non-Specific RNA as a Cofactor in HBV Nucleocapsid Assembly

Solely NTD-driven in vitro assembly of CLPs from CTD-less Cp is both tractable and manipulatable, as elegantly demonstrated by the generation, via an engineered heterodimeric Cp hexamer nucleation seed, of CLPs with defined refillable holes, which may find applications in biotechnology [168]. However, in the biogenesis of authentic pgRNA—P protein containing nucleocapsids assembly of the capsid shell as such represents just one aspect (see above).

A first step towards addressing the role of RNA in HBV capsid assembly was the disassembly of bacterial RNA containing full-length Cp particles into assembly-proficient dimers [108,109,169]. In the absence of other RNAs this even allows to package in vitro transcribed pgRNA, however and in contrast to viral replication, without detectable discrimination against similarly sized heterologous RNAs [108]. Also other polyanions [169,170] as well as single-stranded (ss) DNA [171] and some double-stranded (ds) RNA [172] have been packaged in vitro into regular CLPs but not dsDNA [171], likely due to the stiffness of dsDNA. Similarly, during HBV infection the growing ds-DNA from capsid-internal reverse transcription could increasingly stress the capsid from within and thus contribute to mature rcDNA release into the nucleus (see Figure 6). In line with this view is the selective sensitivity of mature DNA containing but not immature ss-DNA containing nucleocapsids against freezing in the presence of an intercalating dye [173].

In contrast, already the harsher conditions required to disassemble RNA-containing versus empty CLPs indicate that ssRNA stabilizes the nucleocapsid by the attractive electrostatic interactions with the Cp CTDs. These extra interactions also provide access to additional and/or alternative nucleation seeds, beyond the protein–protein interaction governed assembly of physically or functionally CTD-less (by CTD phosphorylation) Cp (Figure 3B), and, thus, are expected to enhance the efficacy of assembly. As HBV nucleocapsid assembly in vivo occurs in the cytoplasm, i.e., in the presence of different viral and a huge excess of cellular RNAs, this bears a high risk for sequestering Cp into replication-incompetent nucleocapsids harboring non-specific RNA, a problem generally shared by viruses which do not employ motor-driven genome packaging into preassembled capsids, such as the (+)-strand ssRNA viruses and retroviruses. Classically, specificity in packaging their genomic RNAs has been explained by the selective presence in those RNAs of a packaging signal (PS) with particularly high affinity to the respective capsid protein; a prototype is the RNA-mediated assembly of the bacteriophage MS2 nucleocapsid [174]. The MS2 coat protein binds particularly well to the MS2 operator, a 19 nt RNA stem-loop, thus nucleating assembly around the genomic RNA. However, newer data suggest more complex scenarios which can account for the often only moderately higher affinity of a capsid protein to the cognate versus non-specific RNA [175]. Recent proposals include the existence of additional packaging-signal-like stem-loop elements (“secondary packaging signals”) throughout the genomic RNA which also bind to the capsid protein and thus cooperatively contribute to the specificity of viral RNA packaging; this mechanism likely applies to several ssRNA viruses, including picornaviruses. Remarkably, also HBV pgRNA contains a few small stem-loops that feature an RGAG motif (R = purine) and bind more strongly to the CTD than random RNA; indeed, they promote in vitro CLP assembly from full-length Cp under conditions that do not lead to protein-only assembly [176,177]. However, more than an auxiliary role of such secondary packaging signals appears not very likely because authentic pgRNA encapsidation is strictly dependent on binding of the viral polymerase to the ε signal [57] (see Figure 3A), and this interaction is necessary and sufficient to mediate encapsidation of unrelated RNAs engineered to carry a 5′ proximal ε element [178,179]. However, unless the RNA binding capacity of Cp is dampened by phosphorylation (see above), RNAs with high CTD affinity might be attractive to deliberately sequester Cp into nonproductive nucleocapsids in HBV infected cells. Conversely, in vitro reconstitution of Cp assembly around the authentic pgRNA-P protein complex remains a highly desirable but demanding goal, including due to the exceptional difficulties in recombinant expression of functional hepadnaviral P proteins [58] yet also its multifactorial nature in vivo.

## 5. Targeting HBV Capsid Dynamics

As emphasized above dynamic changes in HBV Cp and capsid status on several levels are crucial for productive progression through the viral infectious cycle. Hence interfering with any of these dynamics should have antiviral potential although targeting structural proteins is thus far not a widely considered antiviral strategy.

### 5.1. Viral Structural Proteins as Therapeutic Targets

The classical targets for anti-infectives are enzymes of a pathogen. Enzymes are, by definition, capable of multiple turn-over, hence few molecules can catalyze many reactions and inhibiting the enzyme requires little drug but has a large effect. In addition, the natural substrates of viral enzymes are straightforward templates for inhibitor development. Structural proteins have more rarely been considered as antiviral targets. While their usual lack of human homologs predicts lower off-target risks their number per virus particle commonly exceeds that of viral enzymes by far. Hence, a stoichiometrically acting antiviral targeting each structural subunit individually would require unreasonably high dosing. However, in a highly symmetrical viral capsid the individual subunits are not independent, i.e., a perturbation at one site, e.g., by drug binding, can affect distant site in the same subunit yet also in more remote subunits; this is the principle of allostery. In essence, it might be sufficient to target a fraction of subunits, or Cp dimers for HBV, to drastically alter the particle’s properties. One effect could be to perturb the inter-subunit contacts such that assembly of regular particles is hindered or inhibited; less obviously, drug binding to a seemingly regular particle could freeze the dynamics of this particle, i.e., trap it in a state from where it cannot progress further in the replication cycle. Classic examples of this type are the “WIN” compounds (developed at Sterling-Winthrop), such as pleconaril, which bind to a pocket in the Vp1 protein of many picornaviruses (e.g., human rhinovirus, and polivirus) and prevent the release of viral RNA and Vp4 upon contact with the cellular receptor. Pleconaril and alike compounds eventually showed insufficient on-target efficacy and low-resistance barriers in vivo, yet new improved drugs with a similar MoA are under development [180]. Also compounds targeting the non-icosahedral capsid of human immunodeficiency virus 1 (HIV-1) are being investigated [181,182,183]. Over the last years, however, compounds interfering with the dynamics of the HBV nucleocapsid have become a paradigm for the whole field, as also attested by the founding in 2005 of a biotech company, now Assembly Biosciences, Inc., with a primary focus on this topic. However, also many other pharmaceutical companies are engaged in the quest for new, capsid assembly modulating anti-HBV therapeutics.

### 5.2. Discovery of the First and Newer HBV Cp Targeting Compounds

The different terminologies such as CAM, CpAM, CAE, or CI, and the non-uniform use of subcategories such as class-I and class-II compounds caused much confusion. Consensus is emerging to use the acronym CAM which may be appended with an extra letter to specify the phenotypic impact on assembly. The two major established phenotypes are the induction of non-capsid-like *aberrant* multimers by CAM-A compounds, and the induction of apparently regularly shaped but genome-less *empty* capsids by CAM-E compounds. We will here use the CAM-A vs. CAM-E terminology, although the respective phenotypes are not invariant but can be affected by the occupancy of drug binding sites on the particle. Moreover, based on the multiple functions of HBV Cp and the nucleocapsid one could well envisage additional distinct antiviral phenotypes, e.g., formation of seemingly regular genome-containing nucleocapsids which are, however, blocked in genome maturation or controlled mature genome release into the nucleus.

The prototypic CAM-E and CAM-A chemotypes of the phenylpropenamide (PPA) and heteroaryldihydropyrimidine (HAP) class were discovered some 20 years ago as non-nucleosidic inhibitors of HBV replication in cell culture, termed AT-61 and AT-130 [184,185], and BAY41-4109 [186]. The PPAs were found to prevent pgRNA packaging without a major impact on Cp levels and/or capsid shape; the HAP compound depleted Cp and capsids from the cells, supposedly by inducing their proteasomal degradation [187]. Either mechanism disrupted capsid-internal reverse transcription of HBV pgRNA. A third chemotype are the sulfamoylbenzamides (SBAs) which belong to the CAM-E type [188]. Newer additions are sulfamoylpyrroloamides (SPAs), glyoxamoylpyrroloxamides (GLPs) [20,189], and dibenzothiazepins (DBTs). Besides cell-based assays requiring a posteriori definition of MoA, biochemical assays are available that directly monitor the impact of compounds on in vitro assembly [190], or which exploit meanwhile available structural data for in silico docking screens [191]. Hence, there is a growing list of CAMs, many of which are described in a recent review that includes numerous chemical structures [20], yet the currently known MoAs all converge at a common binding site, the inter-dimer interface of the capsid (Figure 7).

### 5.3. Towards Understanding the Mechanisms of CAM Action

Many biochemical and biophysical in vitro methods and cell culture assays established to study HBV assembly as such have been adapted to elucidate how CAMs achieve their anti-HBV effects. In vitro assembly, mostly of recombinant CTD-less Cp, further illuminated the initial in-cell observations. Prevention of closed capsid shell formation by CAM-A compounds obviously perturbs proper replication. CAM-E compounds, seemingly not affecting the final CLP structures (but see below), accelerated assembly rates, suggesting that their antiviral activity in vivo is premature capsid assembly, probably by switching authentic nucleation by the pgRNA/P protein complex to drug-induced Cp-only assembly [192].

#### 5.3.1. Biophysical/Structural Characterization of HBV Cp-CAM Interactions

**Figure 9:**
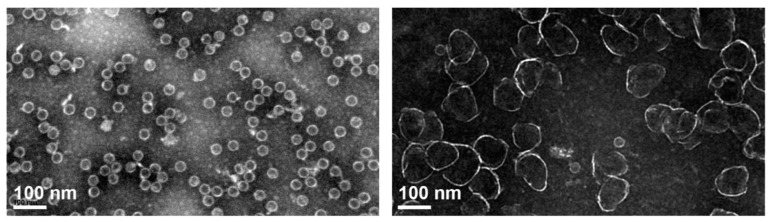
Typical impact of a CAM-A compound on in vitro assembly of Cp149. *E. coli* derived Cp149 CLPs were disassembled and reassembled in the absence (left) or presence (right) of an experimental CAM-A compound, JNJ 890, at a molar Cp:compound ratio of 2:1. Samples were analyzed by negative staining EM and are shown at identical magnification. Formation of large flat aggregates is a hallmark of CAM-A compounds; CAM-E compounds would not produce a particle phenotype that can be distinguished by this technique. Figure courtesy of Lauriane Lecoq and Anja Böckmann.

Numerous HAP CAM-A compounds also accelerate assembly kinetics [193] at low concentration but induce formation of large nonicosahedral arrays of Cp at stoichiometric concentrations [193]; an example of such misdirected assembly is shown in Figure 9 which compares negative staining EM data of Cp149 reassembly in the absence vs. presence of HAP compound JNJ-890 [194].

Structural studies of CLPs with bound CAMs provided more direct information. The assumed tolerance of the capsid structure towards CAM-E compounds suggested by ensemble measurements was challenged by single particle analysis which revealed that AT-130 induced formation of empty as well as only partially completed particles [195]. In vivo such holes would dramatically change accessibility of the capsid interior to macromolecules, amongst them kinases and phosphatases, nucleases, or components of the innate immune system, all of which might represent additional layers of antiviral activity.

**Figure 10:**
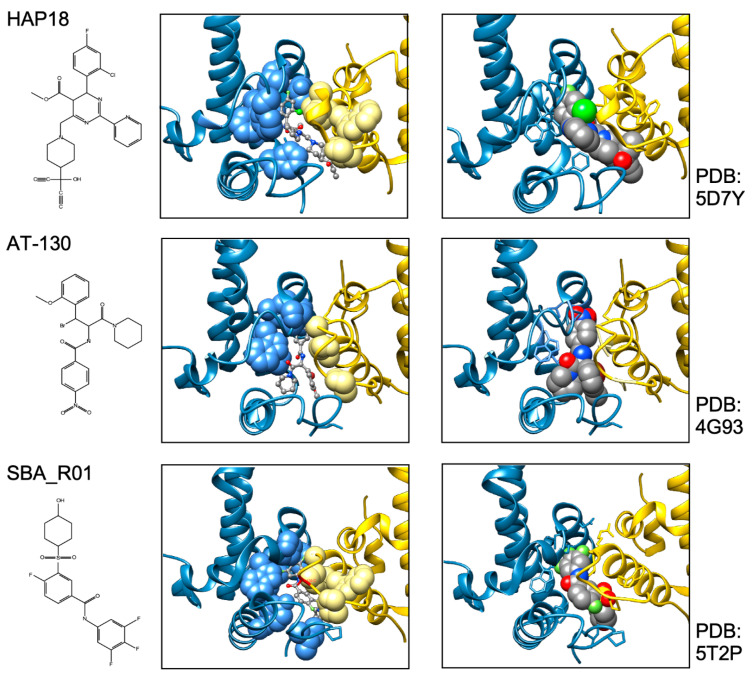
Different CAMs share a common binding pocket yet display non-identical binding modes. All currently known CAMs bind in the HAP pocket formed at the Cp inter-dimer interface with one Cp subunit (in blue) providing most contacts, and the second subunit (in gold) “capping” the bound compound. The compounds represent the classical chemotypes of HAP (HAP18), phenylpropenamide (AT-130) and sulfamoylbenzamidine (SBA_R01). Shown from left to right are the name and chemical formula of the respective CAM; a close-up of the HAP pocket with the most relevant protein residues as spheres and the CAM as ball-and-stick model; and the same view but some relevant protein residues as sticks and the CAM atoms as spheres. The corresponding PDB entries are given on the right. Note that despite their different chemical structure all three CAMs fit snuggly into the binding pocket yet engage both common and distinct Cp residues. This correlates with overlapping but non-identical resistance profiles and addresses different pressure points in the capsid shell which, via allostery, results in different overall responses.

Regarding CAM-A compounds an inherent difficulty is their global impact on capsid structure. Notably, ssNMR should be suitable to characterize these interactions even in non-regular multimers [152] whereas cryo-EM and X-ray crystallography require technical adaptations. One approach [196] utilized a Cp149 derivative with the authentic cysteines replaced by alanines and an extra Cys added after residue 149 (C150) which can form a luminal network of capsid stabilizing disulfide bridges. Here HAP1 induced global changes in the capsid, such as prolapsing of the five-fold vertices and flattening of the quasi-sixfold vertices. The putative HAP1-binding sites were proposed to reside at interdimer contacts between the B and C but not the A and D contacts (see Figure 4C) where the compound would act as a “hydrophobic glue” to strengthen the interaction between the two subunits. This has been confirmed by higher resolution cryo-EM studies showing that HAP13, and most likely other CAMs, can bind to, distort, and even disrupt intact capsids [131]; notably, HAP13 also targeted the B-C dimer contacts in T = 3 capsids, implying that assembly into these alternative morphotype would not be a drug-escape mechanism. Another approach employed the assembly-incompetent Cp mutant Y132A which readily crystallizes, including as trimer of dimers, circumventing formation of nonicosahedral particles [197,198,199]; notably, the crystal contacts are reminiscent of but nonidentical to those in capsids. Still, in these and further studies [20] the “HAP binding pocket” resides at the inter-dimer contacts, and also the other current CAM chemotypes bind to this pocket (Figure 10).

Why then are CAM-A and CAM-E phenotypes different? At least one explanation is that the modes of binding are similar but not identical, i.e., the contributions of individual Cp residues to binding a specific CAM can differ, and so can their impact on association kinetics as well as capsid geometry (Figure 10). This is also manifest by overlapping but nonidentical resistance profiles [200]. In view of the capsid as an allosteric linkage assembly with options for intrasubunit, intradimer, and interdimer conformational crosstalk [149,196], supported by in silico data [161], such differences in CAM action are not really surprising. For instance, the CAM-E AT-130 and the CAM-A HAP1 expanded the capsid, while another CAM-A, HAP18, slightly contracted the particle [201]. Many CAMs bind to the interface between B and C subunits of neighboring AB and CD dimers, i.e., around the quasi-six-fold vertices at the two-fold symmetry axes (see Figure 4C) but not to the pentameric contacts around the five-fold axes [131]. For a HAP13 derivative this was shown to flatten the hexameric and bulge out the pentameric vertices, increasing the particle diameter [131]. Such data underscore how the specific CAM-Cp contacts can differentially affect morphological phenotypes, including the hexagonal sheet or tube structures seen with some CAM-A compounds [193,202]. Notably, the dibenzothiazepine DBT1 (related to ABI H0731/Vebicorvir [203]) occupies less volume at the binding site than most other CAMs and thus can bind to all four quasiequivalent interdimer interfaces. Also, DBT1 accelerates assembly and locally stabilizes interdimer contacts, yet eventually causes capsids to rupture. Plausibly, increasing occupancy puts the capsid as a whole under increasing strain which is relieved by the release of subunits from the shell [204]. Therapeutically, mature dsDNA containing nucleocapsids which are already stressed from within (Figure 6) may be particularly vulnerable to such a mechanism which might lead to untimely accessibility of the viral genome.

In sum do the biophysical data show that all current CAMs bind to essentially the same pocket at the interdimer interface; a comprehensive list of available high-resolution HBV capsid and Cp Y132A hexamer structures with bound CAMs is given in ref. [20]. They all locally stabilize the interaction and accelerate assembly, but, depending on the detailed binding mode, they can have differential impacts on the capsid structure and stability. For classic CAM-E compounds the kinetic effects seem to dominate over structural perturbations, resulting in seemingly normal capsids but overruling the role of the pgRNA-P protein complex as the natural nucleation seed (“over-initiation”). Conversely, for classic CAM-A compounds structural perturbation appears more important. However, with the discovery of ever more CAM chemotypes and derivatives thereof a whole spectrum of phenotypes can be expected which will also depend on drug concentration and binding site occupancy. All these effects should interfere with proper progression of the viral lifecycle.

#### 5.3.2. Preclinical Assessment of CAM Anti-HBV Activity

Though already complex the HBV infectious cycle scheme (Figure 2) largely ignores the crucial role of host factors in nearly all steps [205] and their mutual interdependencies. Just one example are the multiple protein kinases and phosphatases, whose varying intracellular concentrations and localizations can be expected to influence the probability of Cp phosphorylation, and in consequence nucleic acid binding and assembly pathways. Abundant cellular chaperones are known to mediate initiation of HBV reverse transcription [206] but could also generally affect assembly kinetics by trapping non-assembled Cp or assembly intermediates, which in turn would impact phosphorylation and non-specific RNA packaging, and thus also the Cp dimer concentration available for productive nucleocapsid assembly. 

Obviously, in vitro systems provide instrumental concepts on what could happen in a cell but experimental validation is indispensable. Of course, this also holds for the pharmacological aspects of the compounds themselves which we do not consider here. Different from many other human viruses, propagation of HBV in cell culture has long been hampered by the virus’ restricted host range (humans and great apes) and strict liver tropism, limiting experimental infection to primary human hepatocytes or differentiated cells of a single human hepatic cell line, HepaRG. A simpler and widely used substitute was transfection of cloned HBV DNA into the human hepatocyte cell lines HepG2 and Huh7 which circumvents the early infection steps from entry to cccDNA formation while progeny virion formation is supported. The discovery of NTCP as HBV entry receptor has meanwhile allowed to engineer NTCP-expressing HepG2 cells that are susceptible to HBV infection, albeit infection efficiency is very limited, with no net amplification of the input virus. There are also no convenient small animal models for infection with human HBV; in vivo infection of tree shrews is inefficient [207] and mice, by necessity immunodeficient, need to be xenotransplanted with human hepatocytes to become susceptible. Improvements are under way on various levels [208,209,210] but not yet widely available. Hence the mainstays in early preclinical anti-HBV drug development are still transiently HBV transfected or stably HBV producing hepatoma cells such as HepG2.2.15 [211], HepAD38 [212], and HepG2.117 [213], followed by infection experiments in HepG2-NTCP and/or HepaRG cells, primary human hepatocytes, and human liver chimeric mice. Not infection-based in vivo techniques include transduction with adeno-associated virus (AAV) vectored HBV genomes (AAV-HBV) and transfection by hydrodynamic tail-vein injection. Classical animal model viruses for in vivo infection such as DHBV [214] and woodchuck hepatitis virus (WHV) [215] have been highly valuable in the evaluation of NUCs due to their P proteins’ shared enzymatic features. However, despite its overall similarity to HBV Cp WHV Cp differs at various positions in the HAP binding pocket [162], and the structure of DHBV Cp differs even more fundamentally [51]. As experimental infection of chimpanzees with human HBV has been banned clinical trials are mandatory to assess the safety (phase 1) and then dosing and efficacy (phases 2a, 2b) of drug candidates. Phase 3 is intended to confirm safety and efficacy in larger patient cohorts. 

As of yet none of the current anti-HBV CAM compounds is in phase 3 trials but several advanced into phase 2. Below we generally summarize phenotypic in-cell results reported for different CAMs without discussing/commenting on the specific test system they were derived from. However, readers should be aware that promising preclinical data often do not translate into the clinical trial level, owing to inacceptable safety profiles as well as insufficient in vivo efficacy. Regarding mechanistic inferences from cell culture assays it is crucial to consider the specific experimental schemes applied. For instance, reduced cccDNA formation upon HBV infection in the presence of a compound mimics a prophylactic scenario that may not be relevant when cccDNA is already established. However, this notion depends crucially on whether or not the cccDNA pool in CHB is static on the molecular level or is dynamically replenished by de novo synthesis (and, if so, by intracellular recycling or by de novo infection). This also affects potential combination therapies and thus remains one of the most urgent questions to be answered in the field. 

Altogether, and in line with the multiple functions of Cp and the capsid throughout the viral life cycle, CAMs have shown interference at multiple points of the cycle, although the required effective concentrations for 50% inhibition (EC_50_ values) may widely differ for each target step. Given the central role of the HBV capsid as replication compartment and the ability of CAMs to prevent proper assembly it is not surprising that both CAM-E and CAM-A compounds reduce formation of new viral DNA, either by preventing encapsidation of the pgRNA template, or by disrupting the closed environment provided by an intact capsid shell and aggregation of the irregular Cp polymers [216], possibly followed by degradation via autophagy or cell death due to cytotoxic effects, as in many protein misfolding diseases [217]. That Cp mutants mimicking a filled HAP pocket showed reduced DNA synthesis [218] suggests a crucial role for flexibility in the capsid shell during reverse transcription, although this may not easily be distinguished from selective destabilization of mature DNA containing nucleocapsids [219]. At any rate is the reduction, compared to control, of intracellular and extracellular HBV DNA levels a prime parameter for a CAM’s anti-HBV activity. Intracellular DNA levels may be subcategorized into cytoplasmic nucleocapsid-borne and nuclear DNAs, and extracellular DNAs in those in secreted enveloped virions proper and/or other viral particles released into the culture media. As a reference, EC_50_ values for reducing cytoplasmic nucleocapsid-borne HBV DNA with the prototypic PPA, SBA, and HAP compounds were in the range of 200–800 nM. For newer compounds this has been lowered into the low nM range. DNA reductions of up to 3–4 log10 levels have been reported, although this also depends on the dose and duration of treatment. Expectedly, production of fewer genome-containing nucleocapsids inside the cell largely correlates with lower extracellular DNA levels; conceptually a CAM could also affect the efficiency of nucleocapsid envelopment and secretion but this has not systematically been investigated. 

Using infection assays, further potential activities of CAMs, including on cccDNA generation, can be addressed. For instance, the CAM-E compounds JNJ-827 and JNJ-6379 as well as the CAM-A JNJ-890 prevented cccDNA formation when given prior to though not post infection while extracellular DNA was reduced in either case [194,220]; similar data were reported for the CAM-A HAP_R01 [221]. Hence CAMs can likely prevent the incoming nucleocapsid from proper functioning in nuclear transport and/or timely disintegration and genome release, although the EC_50_ values were >10-fold higher than for DNA replication inhibition. In line with the likely intrinsic destabilization of nucleocapsids by progressing internal dsDNA synthesis, nucleocapsids containing mature DNA genomes appear particularly sensitive to CAM-mediated disassembly [219,220] which could be relevant both early (incoming nucleocapsids) and late in infection (newly produced nucleocapsids). Reduced cccDNA formation yields fewer transcription templates and hence fewer viral antigens, all of which have experimentally been confirmed (see [20] and references therein). Additional effects reported were reductions in extracellular HBV RNAs [194,222,223] by CAM-E compounds, likely via their inhibition of pgRNA encapsidation; especially JNJ-827 also reduced secreted HBeAg [194] by a posttranscriptional mechanism. Given the distinct structure of HBeAg (Figure 5) the compound may already interact with incompletely ER-translocated precore protein prior to formation of the decisive C-7/C61 disulfide bond, or induce inter-HBeAg dimer interactions that result in aggregation and perhaps degradation, similar to the impact of CAM-A compounds on Cp; this apparently includes a relocalization of nuclear Cp to promyelocytic leukemia (PML) protein nuclear bodies which are hubs for protein modification and degradation [224]. However, which of these activities turn out to be the therapeutically most relevant requires clinical evaluation. 

#### 5.3.3. CAMs in Clinical Trials

A survey of CAMs in previous and ongoing clinical trials is given in Table 1. Below we discuss some of these compounds in more detail. Clinical development of the prototypic HAP compound BAY41-4109 was discontinued early on, supposedly due to nephrotoxicity. One of the first CAMs to successfully pass phase 1 was the CAM-E NVR 3-778 [225] which had shown anti-HBV efficacy in cell-culture studies [226], in mice with humanized livers [223], and also in patients with chronic HBV infection patients, especially when combined with pegylated type I IFN (pegIFN) [227]. These initial trials demonstrated the validity of the CAM approach and also the option of its combination with orthogonal therapeutic strategies (see below). Eventually, suboptimal potency compared to approved NUCs (HBV DNA reductions of ~1.4 log10 IU/mL vs. ≥2.5 log10 IU/mL after 28 days of treatment), high pill burden and potential adverse effects halted further development of NVR 3-778.

The sulfamoylpyrrolamide JNJ-56136379 (short JNJ 6379) is a CAM-E [220] that was well tolerated and in CHB patients showed substantial anti-HBV activity in phase 1, including mean 2.7 log10 reductions in serum HBV DNA at the end of treatment (EoT) at day 29; in a third of the patients HBV DNA was below the lower limit of quantification [228]. Meanwhile, JNJ 6379 proceeded into 48 week phase 2 trials initially comprising monotherapy and several combination arms (JADE study NCT03361956). Different from the four-week treatment in phase 1 [247] drug-associated viral breakthrough was seen in some patients after 24 weeks of monotherapy but not in combination with a NUC [248]; the combination also achieved higher HBV suppression than NUC alone [229], especially in treatment-naïve HBeAg^+^ CHB patients. Moreover, a higher fraction of these patients showed more pronounced (though still modest) reductions in HBsAg and HBeAg levels, often associated with flares of elevated serum alanine transaminase (ALT) levels, in line with liver inflammation. This may indicate toxicity yet also a therapeutically beneficial reactivation of the immune system whose insufficient activity, or exhaustion, is a hallmark of CHB. Monotherapy with JNJ 6379 is not further developed but several phase 2 trials employing different combination arms are ongoing. A next generation CAM-E earlier in development is JNJ-0440 [230].

ABI-H0731 (Vebicorvir) is a dibenzothiazepine-based CAM-E that proceeded into phase 2 trials after promising phase 1 data, including up to 2.8 log10 reductions in HBV DNA after 28 days of treatment and an apparently low potential for resistance, although one patient with a preexisting Cp T109M mutation showed enrichment of the mutant and a reduced anti-HBV response [231]. In follow-up phase 2 studies combinations with NUC also achieved quicker and stronger reductions in HBV DNA and RNA than NUC alone. In a further follow-up a majority of patients receiving Vebicorvir plus NUC for up to 148 weeks had <20 IU/mL of total HBV nucleic and nondetectable to very low HBeAg; also, no enrichment of CAM resistance conferring mutations was observed, likely due to the potent NUC-mediated suppression of genome replication. However, after cessation of therapy no patient cleared HBsAg and all had virological relapse [249,250]. Hence in favor of newer, more potent CAMs, e.g., ABI-H2158 [232] and ABI-H3733 [233], ABI-H0731 plus NUC will not go into phase 3 trials although more complex combinations are still evaluated (see Table 1), a common trend in the field (see Section 5.3.4).

AB-506 [236] is an aminoindane CAM-E which preclinically induced up to 2.8 log10 reductions in HBV DNA but, besides reduced activity against Cp mutant I105T [20], severe liver-related adverse effects in two healthy individuals stopped its further development, again in favor of a more potent newer CAM-E drug, AB-836, of undisclosed structure [237].

GLS4, a direct HAP successor of BAY41-4109, had initially shown less pronounced anti-HBV activity than other CAMs which could be related to its metabolization by CYP3A, one of the cytochrome P450 isoenzymes. Co-application of the CYP inhibitor ritonavir (RTV) enhanced GLS4 trough concentrations and boosted antiviral activity in phase 2 studies, with reported up to 4.4 log10 reductions in HBV DNA without added NUC [242]. Ongoing phase 2b studies evaluate the combination of GLS4/RTV with NUC (entecavir); interim data indicate also here superiority over NUC alone, with transient ALT flares correlating with stronger antigen declines [242]. Another CAM-A HAP compound, RO7049389, also achieved up to > 3 log10 declines in HBV DNA after 28 days of treatment in phase 1 [234,235], and entered phase 2 trials evaluating long-term triple combinations, including with investigational drugs. A further drug in phase 2 is QL-007 about which little detail has been disclosed; notably, a phase 2 study of ABI-H2158, a more potent CAM-E than ABI-H0731, plus NUC has just been discontinued following elevated ALT levels in some patients (https://investor.assemblybio.com/news-releases/news-release-details/assembly-bio-announces-decision-discontinue-clinical-development; accessed on 21 September 2021).

Overall, the available phase 2 data have given promising yet also mixed results. Despite substantial suppression of HBV replication monotherapy with the early generation CAMs is unlikely to achieve sustained virological responses, let alone a cure of CHB. One expectation is that properly designed combination therapies will bring a breakthrough (see below), another relies on several next-generation CAMs with enhanced potency which entered phase 1 trials. Examples include the CAM-E compounds ABI-H3733 [233], AB-836 [237], EDP-514 [238], GST-HG141 [239], VNRX-9945 [240], KL060332 [243], QL-007 (NCT04157257), and ZM-H1505R (NCT04220801), all of which showed EC_50_ values < 27 nM for HBV DNA reduction and, where determined, <200 nM for cccDNA and/or related viral antigen reductions (see Table 1 for the respective Clinical Trial Identifiers). One of the most potent compounds is ALG-001075, administered as its prodrug ALG-000184, with an EC_50_ of ~1 nM for HBV DNA reduction [241]. Preclinical data indicate that even higher potency range can be achieved, as exemplified by ABI-H4334 or ALG-000286, the prodrug of ALG-000111 [244]; additional similarly active CAMs have been identified, such as M-1428 [246], or GLP-26 with its novel glyoxamido pyrrole (GLP) backbone [245]. These new compounds may be sufficiently active for monotherapy application; however, a more likely scenario is the incorporation of CAMs into combination regimens so as to maximize suppression of viral replication, including cccDNA replenishment, and to restimulate the exhausted immune system to jointly restore control. 

#### 5.3.4. CAMs as Combination Therapy Components

The principle underlying combination therapies accounts for the difficulty of pushing inhibition of a pathogen’s function by a single drug to 100%, and the additive and potentially synergistic (though sometimes antagonistic) effects of adding a second or more drugs with alternative targets. The success of this approach is exemplified by “highly active antiretroviral therapy” (HAART) which has largely turned HIV-1 infection into a manageable chronic disease [251,252]. A major aspect of combination therapy is minimization of resistance development since more beneficial mutations per replication cycle are required for the pathogen to cope with the multiple evolutionary pressures (see below). Given the few current therapy choices for HBV infection, CAMs with their orthogonal MoA are thus a highly welcome addition to the established drugs.

Several ongoing combination trials evaluating CAM add-on to NUC and pegIFNα based regimens already support an enhanced antiviral efficacy compared to the respective monotherapy [227,229,242], and triple combination studies of NUC plus pegIFNα plus CAM are under way (see Table 1). Notably, also the classical treatment targets may be further advanced by long-acting NUCs [253,254], non-nucleosidic inhibitors of the P protein functions [255,256], or ways to reduce clearance of pegIFNα in the liver [257].

With the advent of numerous novel DAA, anti-host factor and immune-stimulating approaches the number of possible combinations is ever increasing, with several examples already under evaluation, e.g., CAM plus NUC or pegIFNα plus anti-HBV siRNA [258], viral antigen secretion blockers, or TLR agonists to stimulate innate immunity (see Table 1). For many of these and further combinations the results will be difficult to predict and require experimental evaluation. This will take time and efforts but also comprises opportunities to provide different patient populations with optimally adjusted treatments. However, an immediately visible benefit of combination approaches relates to CAM resistance.

### 5.4. CAM Resistance—A Relevant Therapeutic Issue to Be Addressed

All direct-acting anti-infectives are prone to resistance development which also plagued early anti-HBV RT inhibitors [4]. Host-factor targeting therapies circumvent this problem but are more prone to exert adverse effects [14]. Resistance evolution requires a certain mutation rate and the production of a sufficient number of progeny genomes to generate, select, and fix the proper mutation(s) in the population. Hence a high resistance barrier relies on efficient suppression of genome replication, and a high number of mutations required to achieve resistance while not losing function. This explains the success of entecavir and tenofovir as anti-HBV NUCs. The same criteria hold for CAMs. Regarding replication suppression most CAMs still lag behind NUCs, hence elucidating the Cp sequence space that fits resistance plus proper nucleocapsid functioning is a crucial issue. As propagating HBV in the lab is still not feasible one way to address this question is structure-guided mutagenesis followed by characterization of the respective virus variants in cell culture, the other information source are sequencing data from clinical trials. The high-resolution structures of capsids with bound CAMs (see Section 5.3.1) provide detailed information on the Cp residues involved in CAM binding, and various mutational studies have functionally confirmed their relevance for resistance. For BAY41-4109 up to ~50-fold increased EC_50_ values regarding HBV DNA reduction were observed for the pocket mutants D29G, Y118F and especially T33N which retained about one third the replication capacity of wt-HBV [259]. The similarity but non-identity of binding modes of different CAMs (Figure 10) is also reflected in their resistance profiles; for instance, P25A/S and V124F reduced susceptibility to HAP_R01 but not to the sulfamoylbenzamide SBA_R01 [199]. A new phenotyping assay facilitating comparative analyses of more mutants yielded similar results, indicating that Cp positions 33, 102, 118, and 127 affect both HAP and SBA binding while positions 25 and 109 are more important for HAP than SBA binding [260]. Also here mutation T33N conferred the highest resistance against both CAM chemotypes yet retained about half the replication capacity of wt-HBV. A comprehensive recent study probed 25 HAP pocket residues by 70 single-site substitutions for functionality and resistance to various CAM phenotypes [200]. Most replacements of W102, interacting with many CAMs, abolished Cp assembly; Cp W102Y and Cp W102H formed capsids but exerted much reduced pgRNA encapsidation and viral DNA synthesis. Hence the emergence of single-site resistance at this position, and similarly at Y132, is unlikely. Position T33 tolerated only substitutions with chemically similar residues but these include T33N. Overall, the most relevant resistance conferring mutations identified concerned position P25, T33, I105, and S106. More such studies will help to define potential escape routes for the virus and, specifically, monitor patients for dangerous variants. These will likely not be restricted to single-site mutants because, given the opportunity, even variants with severely hampered replication capacity can regain fitness by compensatory second-site mutations, as is well known from NUC resistance [4]. Notably, natural polymorphisms have been found at HAP pocket positions by searches in HBV databases [261] and a large patient cohort [260], including Y118F and D29A, I105T/L/V and T114I/V but not T33N; the presence or absence of such baseline mutations may thus affect treatment outcome in patients, as seen by 20-fold reduced activity to AB-506 to Cp baseline mutant I105T [262]. In an early 28-day trial with JNJ-6379 half of the patients carried viruses with one or more potentially relevant mutations, including Y118F, I105T, T109M, and T019I, but their therapy response was not generally reduced, despite a detectable enrichment of the Y118F mutant [247]. Importantly, though, 24 week interim data from the JNJ-6379 monotherapy arm of the 48 week JADE study revealed viral breakthrough in several patients which correlated with baseline mutations Y118F and I105T and emergence of the T33N mutation [248]. By contrast, no viral breakthrough or enrichment of CAM resistant variants occurred in the patients from the CAM plus NUC arm of the study. Similar data were seen with ABI-H0731 monotherapy [231] versus a one-year combination treatment with ABI-H0731 plus NUC [263], corroborating the resistance-suppressing effect of more efficient replication inhibition by the drug combination.

ALG-001075, product of the prodrug ALG-000184 represents one of the preclinically most potent CAMs, remained active against Cp mutants F23Y, I105F/T, T109M, Y118F, and T128I, confirming that the resistance barrier of CAMs can be heightened; still, a nearly 30-fold drop in activity against the T33N mutation was observed [241]. Hence success of a potential monotherapy would depend on if, and for how long, the antiviral activity of the compound could suppress the emergence of this or related mutations. Conversely, these data support the further development of combination therapy approaches.

## 6. Conclusions, Open Questions, and Perspectives

Small molecules targeting HBV nucleocapsid assembly have proven their ability to interfere with HBV replication in CHB patients. They induce significant drops in HBV viremia, viral antigens, and even reduce cccDNA levels by targeting different functions of Cp and the assembled capsid. The efficacy of viral DNA suppression by recent CAMs is approaching that of current first-line NUCs, hence capsid assembly modulation is clearly a valid antiviral strategy. However, the as yet few pertinent clinical data indicate viral rebound after cessation of therapy, even in first combination trials with a NUC. Hence at present CAMs appear as a highly valuable addition but not a fundamentally superior substitute for current NUC monotherapy. Nonetheless, it is still early days and prospects for significant improvements are good even though more virological, pharmaceutical and clinical efforts will be needed for a final judgement, owing to numerous knowledge gaps on the functional and conformational dynamics of HBV Cp and its assembly products.

There is still a huge gap between the high-resolution data available for Cp-NTD-only mediated in vitro assembly versus the complexity of authentic nucleocapsid assembly in vivo. There, numerous additional interactions are involved and their relative influence is modulated by the specific conditions in an individual cell. For instance, assembly depends on the available Cp concentration which depends on intracellular localization which, in turn, is affected by assembly status. CTD-nucleic acid interactions promote assembly but depend on the phosphorylation status which in turn is affected by the available protein kinases and phosphatases and accessibility of their Cp target sites which again depends on assembly status. Moreover, maturation of the packaged genome will affect capsid stability, and this spiral will go on if further host factors and the CAM activities are also considered; an example is the synergistic disruption of capsids by a HAP compound and importin β [264]. Eventually it may be possible to theoretically model this multifactorial process and predict how individual parameters impact the outcome but at present experimental approaches will be indispensable to further substantiate and evolve models like that in Figure 6. Improved cell culture techniques should make more authentic nucleocapsid samples amenable to high-resolution biophysical analyses; a prime example are nucleocapsids in defined stages of genome maturation (Figure 3A).

More knowledge is also required on the specific recognition of the pgRNA-P protein complex by Cp, the exact role of phosphorylation/dephosphorylation in stowing the different genome forms, and the mechanics of capsid–internal reverse transcription. Particularly enigmatic are the movements of nucleic acids, P protein, and CTD domains that must accompany polymerization (Figure 3A). Is P static relative to the capsid shell and pulls the actual template sequences through its active site, or does it move along the nucleic acid templates? How could this be coordinated with capsid–internal kinases and phosphatases? Does the capsid actively support the whole process by iterative conformational changes, either locally or via allostery, throughout the capsid shell?

A related issue is whether a newly formed nucleocapsid is really identical to the incoming nucleocapsid from the infecting virus, as implied in Figure 2 and other schemes of the HBV life-cycle. If not, due to their different histories, this could well affect their nuclear transport capacity as well as CAM responsiveness.

These are not only academic questions as they also relate to the interpretation of capsid-CAM interactions in vivo. Different capsid types, such as genome-containing versus empty, could well interact differently with one CAM. In a therapeutic setting empty capsids seem less relevant targets which may even sequester drugs from targeting the real nucleocapsid. Hence the inclusion of different capsid types would provide relevant new information for CAM optimization. A first example could be the application of solid-state NMR which can characterize CAM-A binding in irregular multimers, without need for workarounds such as non-wildtype Cp variants (see Section 5.3.1). The eventual overall aim should be an increasing harmonization of in vitro and incell data.

Also, the clinical data comprise unresolved basic issues, including the causes and consequences of the liver damage-indicating ALT flares seen in several CAM trials (see Section 5.3.3). Both may be related to the ill-defined post-treatment fate of HBV-positive cells where CAM-A, and likely also CAM-E, compounds mediate accumulation of capsid-like and irregular particles [216]. Are those cells doomed to die, either spontaneously, or via killing by reactivated virus-specific immune cells? Is such cell-death eventually beneficial or detrimental for the host organism? Are HBV-negative cells also affected by off-target toxicity?

Most relevant for optimizing CAM combination therapies aimed at curing CHB is to disentangle how the long-lived cccDNA reservoir in the infected liver is maintained. Longevity on the individual cccDNA molecule level would leave little prospect for CAM-mediated interruption of cccDNA neogenesis while cccDNA turnover would make this a valid option. Defining the contributions of intracellular recycling versus de novo infection to replenishment [36] would further inform combination designs, e.g., inclusion of drugs with intracellular impact such as pegIFNα in the first case and an entry inhibitor, such as bulevirtide, in the second. Obviously solving these questions would very much broaden the basis for evidence-based combination therapies.

Not the least, advances in medicinal chemistry hold promise for new CAM molecules with improved pharmacokinetics, pharmacodynamics, and target-organ specificity and potency.

Moreover, HBV Cp and the capsid provide further pharmacologically unexplored interaction sites whose occupancy by specific ligands can be expected to have similar allosteric consequences as CAMs. One example are the spike tips where specifically binding peptides have been identified [163], another is a hydrophobic pocket in the spike base where specifically binding alkyl phenoxypolyethoxylethanol compounds induce a distinct conformational change [73].

Lastly we note that CAMs lend themselves as building blocks for PROteolysis TArgeting Chimeras (PROTACs), heterobifunctional small molecules combining a constant E3 ubiquitin ligase recruiting moiety with a specific targeting module, and, thus, induce the target’s ubiquitylation and subsequent proteasomal degradation [265].

In sum, after decades with peg-IFNa and NUCs as the only treatment options for CHB novel antivirals are paving the way for a much broader attack on the HBV infectious cycle. Interference with HBV nucleocapsid dynamics is likely to play an important role in this battle even though the path to an HBV cure may still be long.

## Figures and Tables

**Table 1:** Anti-Hbv activities of CAMs in preclinical and clinical evaluation.

CAM/*CAM Type*	Chemo- Type	Phase	In Vitro EC_50_ [nM] ^a^			Mean log10 Reduction at End of Treatment Period (TP)	Trial ID ^c^	Combination Trial Agents ^d^	Sponsor	Refs.
			**DNA_ic_/DNA_ec_**	**RNA_ic-t_/pgRNA_ic/_HBs**	**cccDNA**	**DNA/RNA (TP) ^b^**				
*Prototypical CAMs*										
**AT-130/*E***	PPA	P/C	~200–2500/ND	ND/ND/ND	ND	NA	NA			[185]
**DVR-23/*E***	SBA	P/C	779/ND	ND/ND/ND	ND	NA	NA			[188]
**BAY 41-4109/*A***	HAP	1; DC	33–276/ND	ND/ND/ND	ND	ND	ND	NA	AiCuris	[186]
*CAMs in recent and ongoing trials*										
**NVR 3-778/*E***	SBA	1; DC	340/440	3700/ND/4800	ND	1.43/1.42 (28 d)	NCT02401737	pIFN	Novira/J&J	[226,227]
**JNJ-6379/*E***	SPA	2a/b	54–69/102	876/ND/1608	ND	2.70/1.83 (28 d)	NCT04129554, NCT03982186, NCT04667104, NCT04439539	NUC NUC + pIFN NUC + pIFN + JNJ-3989 (a-HBV siRNA)	Janssen/J&J	[220,228,229]
**JNJ-0440/*E***	SBA	1b	12–24/NDR	136/ND/243	ND	3.3/2.6 (28 d)	NCT03439488	NA	Janssen/J&J	[230]
**ABI-H0731 (Vebi-** **corvir)/*E***	DBT	2a; DC	154–307/ND	ND/~2500/~5000	ND	2.8/2.0 (28 d)	NCT03109730, NCT03576066, NCT03577171, NCT04781647, NCT04820686	NUC NUC + pIFN NUC + AB-729 (a-HBV siRNA)	Assembly Biosciences	[203,231]
**ABI-H2158/*E***	ND	2a; DC	22/ND	ND/227/ND	334	2.5/2.2 (14 d)	NCT04398134	NUC	Assembly Biosciences	[232]
**ABI-H3733/*E***	ND	1b	5–12/ND	ND/27–80/43–77	125	TBR	NCT04271592	NA	Assembly Biosciences	[233]
**RO7049389/*A***	HAP	2a	4–62/6.0	ND/ND/~1000	ND	2.86/2.54 (28 d)	NCT03570658, NCT03717064, NCT02952924, NCT04225715	NUC + RO7020531 (*TLR7 agonist*) NUC + RO7445482 (a-HBV siRNA)	Hoffmann-LaRoche	[234,235]
**AB-506/*E***	Amino-indane	1; DC	65–77/ND	ND/ND/1430	ND	~2.2/~2.5 (28 d)	NDR	NA	Arbutus	[236]
**AB-836/*E***	ND	1a/b	2–12/ND	ND/ND/197	175	TBR	NCT04775797	NA	Arbutus	[237]
**EDP-514/*E***	ND	1b	17–27/ND	ND/3–25/35	ND	3.3/2.4 (28 d)	NCT04008004, NCT04470388, NCT04971512	EDP-721 (*HBV RNA destabilizer*)	Enanta	[238]
**GST-HG141/*E***	ND	1a/b	ND/8/ND	ND/ND/ND	ND	TBR	NCT04386915, NCT04868981	NA	Fujian Cosunter	[239]
**VNRX-9945/*E***	ND	1a	ND/2.3–10	ND/ND/90	ND	NA	NCT04845321	NA	Venatorx	[240]
**ALG-000184 (prodrug of ALG-001075)/*E***	ND	1b	0.5–1.4/ND	54/ND/70		2.9/ND (14 d)	NCT04536337	ALG-010133 (*HBsAg release inhibitor*), ALG-125755 (*siRNA*), ALG-020572 (*ASO*), NUC	Aligos	[241]
**QL-007/*ND***	ND	2a	ND	ND	ND	ND	NCT04157699, NCT04157257	NUC	Qilu	see NCT
**ZM-H1505R/*ND***	Pyrazole	1a	ND	ND	ND	NA	NCT04220801	NA	Shanghai Zhimeng	see NCT
**GLS4/RTV *(CYP block)/A***	HAP	2a	ND	ND	ND	4.37/2.47 (24 week)	NCT04147208	NUC	Sunshine Lake	[242]
**KL060332/*A***	HAP	1a	5.0 (n.s.)	ND	ND	NDR	CTR20200985	NA	Kelun-Biotech	[243]
*New CAMs in current preclinical trials*										
**ABI-H4334/*ND***	ND	P/C	0.5/2.4 (n.s.)	ND	ND	NA	NA	NA	Assembly Biosciences	^e^
**ALG-000286 (prodrug of ALG-000111)/*E***	ND	P/C	0.7–0.9/ND	34/ND/42	ND	NA	NA	NA	Aligos	[244]
**GLP-26/*E***	Glyoxamido-pyrrole	P/C	ND/3–4	ND/11/ND	ND	NA	NA	NA	Emory University	[245]
**M-1428/*E***	ND	P/C	“500-fold higher potency vs. NVR 3-778” (n.s.)	ND/ND/ND	ND	NA	NA	NA	Vita-Salute San Raffaele University	[246]

^a^ EC_50_ values [nM] from cell-culture experiments for intracellular (ic) and extracellular (ec) viral DNA; for intracellular total viral RNA (ic-t) or intracellular (ic) pgRNA; for HBsAg. A range of values may result from employment of different assays and/or different cell systems. ^b^ Viral DNA and RNA reductions in patients are given in log10 IU/mL (DNA) and in log10 copies/mL (RNA). ^c^ Clinical trials are registered at cliniclatrials.gov (NCT) or at chinadrugtrials.org (CTR); DC, discontinued clinical development (indicated by orange background). ^d^ Components of ongoing and planned combination therapy trials; pIFN, pegIFNα; ASO, antisense oligonucleotide. General abbreviations: NA, not applicable; ND, no data; and n.s., not specified. ^e^ https://investor.assemblybio.com/news-releases/news-release-details/assembly-bio-selects-fourth-hbv-core-inhibitor-candidate.

## Data Availability

Not applicable—no original data reported.
